# Altered histone abundance as a mode of ovotoxicity during 7,12-dimethylbenz[a]anthracene exposure with additive influence of obesity^†^

**DOI:** 10.1093/biolre/ioad140

**Published:** 2023-10-19

**Authors:** Jaspreet K Rishi, Kelsey Timme, Hunter E White, Karl C Kerns, Aileen F Keating

**Affiliations:** Department of Animal Science, Iowa State University, Ames, Iowa, USA; Department of Animal Science, Iowa State University, Ames, Iowa, USA; Department of Animal Science, Iowa State University, Ames, Iowa, USA; Department of Animal Science, Iowa State University, Ames, Iowa, USA; Department of Animal Science, Iowa State University, Ames, Iowa, USA

**Keywords:** ovary, obesity, DMBA, DNA repair, histones, histone variants, H3.3

## Abstract

Histones are slowly evolving chromatin components and chromatin remodeling can incorporate histone variants differing from canonical histones as an epigenetic modification. Several identified histone variants are involved with the environmental stress-induced DNA damage response (DDR). Mechanisms of DDR in transcriptionally inactive, prophase-arrested oocytes and epigenetic regulation are under-explored in ovarian toxicology. The study objective was to identify ovarian proteomic and histone modifications induced by DMBA exposure and an influence of obesity. Post-pubertal wildtype (KK.Cg-a/a; lean) and agouti (KK.Cg-Ay/J; obese) female mice, were exposed to either corn oil (control; CT) or DMBA (1 mg/kg) for 7d via intraperitoneal injection (*n* = 10/treatment). Ovarian proteome analysis (LC-MS/MS) determined that obesity altered 225 proteins (*P* < 0.05) with histone 3 being the second least abundant (*FC* = −5.98, *P* < 0.05). Histone 4 decreased by 3.33-fold, histone variant H3.3 decreased by 3.05-fold, and H1.2, H1.4 and H1.1(alpha) variants increased by 1.59, 1.90 and 2.01-fold, respectively (*P* < 0.05). DMBA exposure altered 48 proteins in lean mice with no observed alterations in histones or histone variants. In obese mice, DMBA exposure altered 120 proteins and histone 2B abundance increased by 0.30-fold (*P* < 0.05). In DMBA-exposed mice, obesity altered the abundance of 634 proteins. Histones 4, 3 and 2A type 1-F decreased by 4.03, 3.71, 0.43-fold, respectively, whereas histone variant H1.2 and linker histone, H15 increased by 2.72- and 3.07-fold, respectively (*P* < 0.05). Thus, DMBA exposure alters histones and histone variants, and responsivity is more pronounced during obesity, potentially altering ovarian transcriptional regulation.

## Teaser

Exposure to 7,12-dimethylbenz(a) anthracene initiates a more pronounced ovarian proteomic response in obese mice, with specific changes to the abundance of histones and histone variants.

## Graphical Abstract

**Graphical Abstract:**
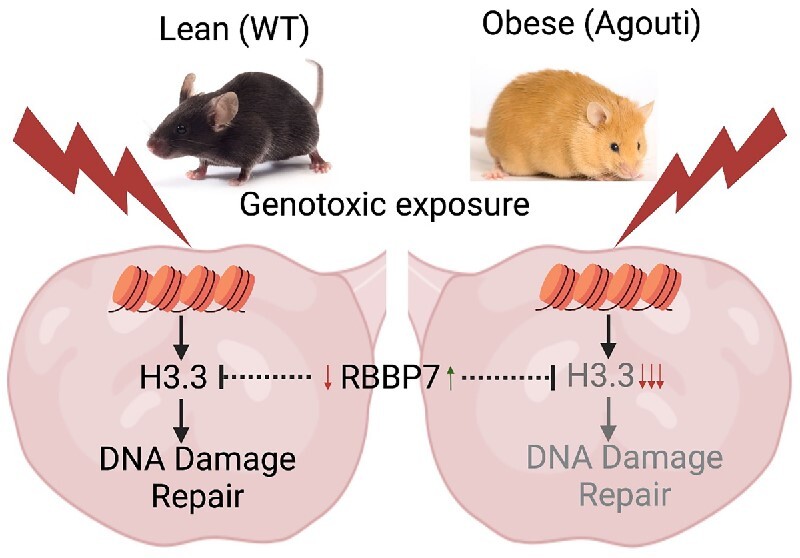

## Introduction

Packaging DNA is a complex task. The ~2 m long eukaryotic DNA [1] must efficiently fit in the microscopic nucleus [2] and must also be selectively accessible for transcription and DNA replication. This elaborate but methodical task is accomplished by lysine and arginine-rich, highly basic proteins, called histones, around which the DNA can be compactly wrapped [3–5]. Together, histones and nucleic acid comprise the nucleosome, the basic unit of chromatin [6]. The core of the nucleosome comprises an octamer of two of each H2a, H2b, H3, and H4 histones, forming a “bead” wrapped by DNA and connected with the linker histone, H1 [7]. The N-terminal tails of histones, particularly those of histones H3 and H4, are available for modifications, including lysine and arginine methylation; lysine acetylation, ubiquitylation, SUMOylation, and ADP-ribosylation; serine, threonine, and tyrosine phosphorylation; and methyl-arginine citrullination [6, 8, 9], to function in processes, including transcription, replication, recombination, and DNA repair [6]. Generally, phosphorylation is associated with chromatin condensation, whereas acetylation is associated with active transcription [9, 10]. However, specific histone modification combinations do not always confer the same biological consequence, which can depend on the cellular context.

Most histones are synthesized during the S phase of the cell cycle for rapid deposition during replication [11]. Occasionally, canonical histones can be replaced by histone variants dynamically, independent of DNA replication, altering chromatin composition and subsequently affecting epigenetic function [11]. Histone variants participate in gene silencing, activation, or compensation [11], and nearly all histone variants are apparently involved in environmental stress-related DNA damage response (DDR) through various mechanisms [12]. Although core histones are highly conserved in eukaryotes [13], a combination of post-translational modifications (PTMs) and incorporation of variant histones can serve as diverse epigenetic marks [11].

Originally associated with affluent nations, obesity is now a global epidemic with a rapid increase observed in recent decades [14], which worsened during the COVID-19 pandemic [15, 16]. Of note is that, in the USA, obesity rates are increasing more rapidly in children and adolescents than in adults [17–19]. Obesity has several negative impacts on female fertility, including hyperandrogenism [20], impaired neuroendocrine functioning [21], reduced ovarian reserve [22], and subfertility and infertility [23]. Obesity is also a genotoxic insult to the ovary as basal levels of DNA damage have been observed in obese mouse ovaries [24] in addition to their having an altered response to ovotoxicant exposures [25–27].

The polycyclic aromatic hydrocarbon (PAH) 7,12-dimethylbenz(a) anthracene (DMBA) is a lipophilic alkylating genotoxicant released during incomplete combustion of organic materials [28, 29]. Cigarette smoke, diesel exhaust, charred meat, overheated cooking oil, petroleum coke or asphalt manufacturing, waste incineration, and wildfires are the PAH sources [30–32], and DMBA is produced during cigarette burning [33] and bread baking [34], and it is an air pollutant [35]. Routes of DMBA exposure are via inhalation and absorption [36], and DMBA is converted in vivo into a genotoxic epoxide metabolite via the ovarian biotransformation enzymes, including microsomal epoxide hydrolase (EPHX1), [37] leading to DNA adduct formation [37, 38]. The genotoxicity of DMBA is well established in tissues, including the ovary [37, 39–42], and exposure to DMBA causes ovarian failure due to the destruction of small and large follicles [37, 43–51].

Elevated ovarian levels of DDR proteins post-DMBA exposure are observed, indicating DNA damage and induction of the DDR response [26, 52]. Considering the role of histone modifications and the potential for histone variants to be involved in DDR, this study investigated the hypothesis that DMBA-induced ovotoxicity involves alterations to the ovarian proteome with specific changes to the abundance of histones and histone variants and that this response would differ due to obesity. Previously, a 14-day exposure to DMBA in 18-week-old lean and obese mice caused follicle loss at all stages of development [24], thus, a shorter DMBA exposure duration was employed to explore mechanisms of ovotoxicity occurring prior to overwhelming follicular damage and loss.

## Methods

### Reagents

The DMBA (CAS #57-97-6), corn oil, dithiothreitol, ethylenediaminetetraacetic acid (EDTA), ethanol, iodoacetamide, liquid nitrogen, mammalian serum, paraformaldehyde, paraffin, phosphate-buffered saline (PBS), PRTC standard, trypsin, tris HCl, Tris Base, and tris-buffered saline were obtained from Sigma-Aldrich (St. Louis, MO). Citrasolv, bicinchoninic acid assay, SlowFade Gold mounting media, and YOYO-1 Iodide (491/509) antibody (catalog # Y3601) were obtained from Thermo Fisher Scientific (Rockfield, IL, USA). Primary antibody against H3.3 (catalog number ab176840) was obtained from Abcam (Boston, MA). Protease/phosphatase inhibitor cocktail (catalog # 5872), Goat anti-Rabbit IgG, and Alexa Fluor 568 (catalog # A-11011) secondary antibody were purchased from Cell Signaling Technology (Danvers, MA).

### Animal exposure and tissue collection

Hyperphagic lean (HPL) female C57Bl6 (KK.Cg-a/a; strain 000664; *n* = 20) wild-type mice and hyperphagic obese (HPO) agouti lethal yellow mice (KK.Cg-Ay/J; strain 002468; *n* = 20) were obtained from Jackson Laboratory (Bar Harbor, ME) and housed two to five per cage at Iowa State University, as approved by the Institutional Animal Care and Use Committee. A 12-h circadian rhythm and 25°C room temperature were maintained. Access to food (2014 Envigo Teklad Global 14% Protein Rodent Maintenance Diet) and water were ad libitum*,* and weekly food intake and body weights were monitored. When an ~30% weight difference was determined between the HPL and HPO mice, they received either corn oil as vehicle control (C) or DMBA (D; 1 mg/Kg/day) via intraperitoneal injection for 7 days. This exposure paradigm and route of exposure was determined to result in a tendency to increase time spent in proestrus and to reduce primordial follicle number in lean mice [53]. In obese mice, DMBA exposure decreased uterine weight and increased primary follicle number [53]. Obese mice had reduced primary, preantral, and corpora lutea number [53]. Thus, there was evidence of ovotoxicity without a complete depletion of ovarian follicles.

Euthanasia occurred at ~10 week of age on Day 2 of diestrus post-dosing (up to 3 days post-cessation of exposure) and the stage of the estrous cycle was monitored via daily vaginal cytological analysis. One ovary from each mouse was frozen in liquid nitrogen and was stored at −80°C for protein analysis. The other ovary from each mouse was fixed in 4% paraformaldehyde overnight, transferred to 70% ethanol, and stored at 4°C for immunostaining.

### Protein isolation, liquid chromatography–tandem mass spectrometry and proteome analysis

Total ovarian protein was isolated using lysis buffer consisting of 50 mM Tris–HCl and 1 mM EDTA (*n* = 5/treatment) to perform liquid chromatography–tandem mass spectrometry (LC–MS/MS). Crude protein samples were reduced using (2*S*,3*S*)-1,4-Bis(sulfanyl)butane-2,3-diol (dithiothreitol (DTT)) followed by cysteine modification by iodoacetamide and overnight digestion with trypsin. Samples were dried in a SpeedVac after adding formic acid, followed by a desalting step and re-drying. An internal control, PRTC standard (Pierce part#88320), was added to the samples. Separating peptides was performed by injecting 5 μg of protein (and 125fmol PRTC) onto the HPLC column (Thermo Scientific EASY nLC-1200). Peptide fragmentation patterns were compared with MASCOT or Sequest HT. Minora Feature Detector node was used to detect LC–MS/MS peaks in the raw data files to map the fragments and using the average-scaling analysis, a theoretical isotope was calculated. New features were created for fragments that were not found in the database. Database for Annotation and Visualization and Integrated Discovery (DAVID 6.8) was used to analyze the bioinformatics data thus generated to compare the abundance of proteins across treatment groups. Altered proteins across treatment groups were compared with the reference database of *Mus musculus* on PANTHER v16.0 to visualize gene ontology and biological pathways. For proteomic pathway analysis all proteins with *P* < 0.1 were included.

### H3.3 protein localization and quantification

Formaldehyde-fixed ovaries were embedded in paraffin, sectioned at 5 μM thickness, and immunofluorescence staining (*n* = 4 ovaries per treatment) was performed to detect H3.3 protein localization and abundance. Slides were warmed in a water bath at 60°C for 30 min, followed by incubation in Citrasolv (3X for 5 min). Tissues were rehydrated in 100% and 70% ETOH 2X for 3 min each and incubated in PBS for 5 min. Antigen retrieval was performed using tris base buffer (pH 9) in a water bath between 95 and 100°C for 30 min. Slides were allowed to cool down for 30 min at room temperature and washed in PBS for 2X for 2 min. Sections were encircled with a hydrophobic PAP pen and blocked with 5% mammalian serum (goat/donkey) for 1 h. This was followed by washing ovarian sections in PBS 3X for 5 min and incubating them in primary antibody (1:1000) overnight at 4°C in a humidified chamber. The next steps were performed in a darkened room. Slides were washed in PBS (3 X 5 min) and incubated in the secondary antibody (1:500) combined with YOYO-1 (1:5000), a cellular DNA stain, for 1 h at room temperature in a humidified chamber. Slides were washed again in PBS (3 X 5 min), and coverslips were added using SlowFade Gold mounting media after blotting. Images were obtained with a Leica DM6 B microscope outfitted with a Lecia K5 camera at the appropriate wavelength for each stain. Images were acquired using Lecia Application Suite X software and the intensity of the H3.3 staining in specific follicle types was analyzed using ImageJ software and normalized by the area of the entire follicle (*n* = 4 sections per ovary from four animals per treatment group). Small follicles were defined as primordial, primary, and secondary follicles. Follicles with a developing or developed antrum were designated as large. The investigator was blinded to the identity of the ovary section treatment.

### Statistical analysis

Unpaired t-test with Welch correction was performed to identify statistical differences using GraphPad Prism 9.0. A *P* value <0.05 (unadjusted) was considered significant and a *P* value <0.1 (unadjusted) was considered as a tendency for statistical significance. The FDR-corrected *P* values (*q*-values) for proteomic data were calculated using the Benjamini–Hochberg method and are included in supplementary tables. Proteins that were altered by treatment comparison at *P* < 0.05 were used for pathway analysis. Two-way analysis of variance (ANOVA) was used to analyze an interaction between obesity and DMBA exposure. For pathway analysis and histone protein profiling, proteins that differed at *P* < 0.1 were included.

## Results

### Effect of obesity on the total ovarian proteome in DMBA-treated mice

**Figure 1:**
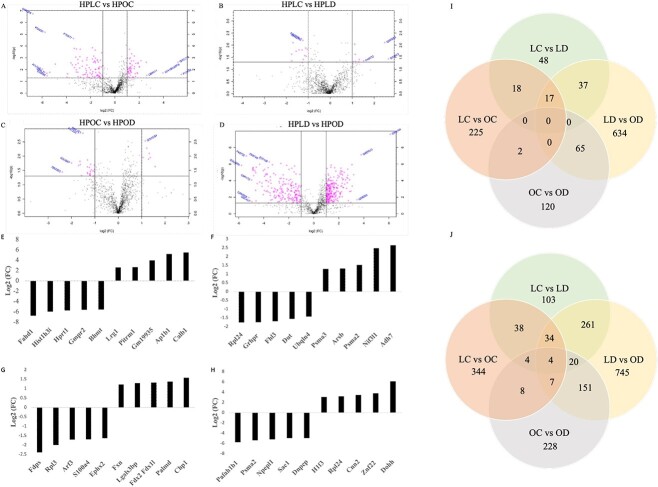
Impact of DMBA on total protein abundance in lean and obese mice ovaries. After 7 days of exposure to vehicle control or DMBA, total ovarian proteome changes were quantified in lean and obese mouse ovaries via LC–MS/MS. (A) HPLC versus HPOC; obesity altered 225 proteins, (B) HPLC versus HPLD; DMBA altered 48 proteins in lean mice, (C) HPOC versus HPOD; DMBA altered 120 proteins in obese mice, and (D) HPLD versus HPOD; DMBA altered 634 proteins in lean versus obese mice. The dots to the right or left of the vertical lines indicate proteins that were altered by a fold change >2. The top five increased and decreased proteins are depicted in the comparisons; (E) HPLC versus HPOC, (F) HPLC versus HPLD, (G) HPOC versus HPOD, and (H) HPLD versus HPOD*. P* < 0.05; *n* = 5. The Venn diagrams summarize numbers of proteins altered independently with treatment and in common between treatments in (I) with cutoff at *P* < 0.05 and in (J) with cutoff at *P* < 0.1).

The total ovarian proteome was analyzed to identify differentially expressed proteins due to DMBA exposure in lean and obese mice (Figure 1). Comparing hyperphagic lean control (HPLC) with hyperphagic obese control (HPOC) ovaries, a total of 1267 proteins were detected, with 225 proteins being differentially abundant (*P* < 0.05), of which 95 proteins were increased and 130 proteins decreased **(**Figure 1A; Supplemental Table 1**)**. Of these, 141 proteins were altered with a fold change of >2 (*P* < 0.05). Pathway analysis was performed via PANTHER to screen for genes involved in female reproduction and DDR pathways (*P* < 0.1). Three proteins were identified with a role in DNA replication, two proteins had predicted involvement with the gonadotropin-releasing hormone receptor pathway, one protein was involved in the p53 DDR pathway, and one other was identified to be associated with the PI3K pathway **(**Supplemental Table 2**)**.

In the next comparison between HPLC and hyperphagic lean DMBA-exposed (HPLD), 1230 proteins were detected, and 48 proteins were altered (*P* < 0.05), of which 19 were increased and 29 decreased **(**Figure 1B; Supplemental Table 3**)**. There were 22 proteins with a fold change of >2 (*P* < 0.05). In lean mice, DMBA treatment did not alter proteins identified to be specifically involved in female reproduction or DDR processes.

In the comparison between HPOC and hyperphagic obese DMBA exposed (HPOD), 120 proteins were altered (*P* < 0.05) of the 1228 proteins detected. Of these, 76 proteins were increased in abundance and 44 were decreased **(**Figure 1C; Supplemental Table 4**)** and 32 proteins were altered with a fold change of >2 (*P* < 0.05). In obese mice, DMBA altered four proteins in the gonadotropin-releasing hormone receptor pathway and two in the p53 pathway **(**Supplemental Table 5**)**.

In the final comparison, between the lean (HPLD) and obese (HPOD) mice exposed to DMBA, 1230 proteins were detected, and almost half of the proteins detected were altered (634 proteins, *P* < 0.05). Of these, 408 were increased and 226 were decreased **(**Figure 1D; Supplemental Table 6**)**. There were 422 proteins with a fold change of >2 (*P* < 0.05). The combination of DMBA exposure with obesity affected two DNA replication pathway proteins, seven proteins in the gonadotropin-releasing hormone receptor pathway, four in the p53 pathway, and two with involvement in the PI3K pathway **(**Supplemental Table 7**)**. Altered proteins are visualized in the volcano plot **(**Figure 1A–D**)** for each treatment comparison and proteins with a fold change of >2 (*P* < 0.05) are represented in pink.

The top five most and least abundant proteins were identified in each treatment comparison **(**Figure 1E–H**)**. Obesity altered acylpyruvase and calbindin to the greatest extent, decreasing the former by 6.7-fold and increasing the latter by 5.5-fold (*P* < 0.05). Interestingly, histone fragment 3 was the second least abundant protein in this comparison (Figure 1E). In lean mice, DMBA decreased TRASH domain-containing protein by 1.8-fold and increased alcohol dehydrogenase class 4 by 6.3-fold (Figure 1F; *P* < 0.05). In obese mice, DMBA decreased farnesyl pyrophosphate synthase by 2.4-fold and increased calcineurin B homologous protein 1 by 1.6-fold (*P* < 0.05). Obesity and DMBA exposure combined decreased the platelet-activating factor acetylhydrolase IB subunit alpha to the greatest level by 5.8-fold and increased deoxyhypusine hydroxylase by 6.1-fold (Figure 1G; *P* < 0.05). Bifunctional epoxide hydrolase (EPHX2) was also one of the least abundant proteins in this comparison. Interestingly, SUMO-activating enzyme subunit 1 (SAE1), which mediates ATP-dependent activation of small ubiquitin-like modifier (SUMO) proteins, was one of the least abundant proteins and TRASH domain-containing protein, which DMBA decreased in lean mice, was increased when obesity was present (Figure 1H).

The total number of proteins altered (*P* < 0.05) and those that tended to be altered (*P* < 0.1) in each treatment comparison, and the proteins that were changed in more than one comparison are illustrated in the Venn diagram **(**Figure 1I and J**).**

### Classification of proteins impacted by DMBA in lean and obese mice ovaries

**Figure 2:**
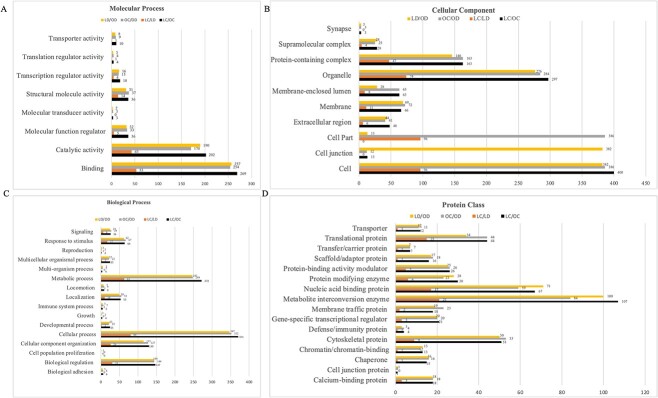
Classification of proteins impacted by DMBA in lean and obese mice ovaries. Proteins identified as altered by treatment or physiological status (*P* < 0.05; *n* = 5) were classified by (A) molecular process, (B) protein class, (C) biological process, and (D) cellular component in four treatment comparisons: HPLC versus HPOC; HPLC versus HPLD; HPOC versus HPOD; and HPLD versus HPOD.

Proteins differentially abundant due to DMBA in lean and obese mice were identified and classified into GO terms based on molecular processes, protein classes, biological processes, and cellular components **(**Figure 2**)** and analyzed using PANTHER software to understand their biological and molecular functions. In the molecular process category (Figure 2A), “binding” and “catalytic activity” were the most represented subcategories of differentially expressed proteins due to both obesity and DMBA. The most represented cellular components were “cell” and “organelle” (Figure 2B). In the biological process category (Figure 2C), “cellular process” and “metabolic process” were the most represented. Protein classes altered by obesity to the greatest extent were “metabolite interconversion enzyme” and “intercellular signal molecule” and those affected greatest by DMBA exposure were “metabolite interconversion enzyme” and “nucleic acid binding” (Figure 2D). The number of proteins altered in each treatment comparison is presented for each subcategory **(**Figure 2**).**

Interactions of differentially expressed proteins were also studied using STRING DB **(**Supplemental Figure 1**)**. Proteins altered by obesity **(**Supplemental Figure 1A**)** basally had 181 nodes (representative of proteins) with 650 edges (representative of interactions). The proteins altered by DMBA in lean mice had 37 nodes and 57 edges **(**Supplemental Figure 1B**)**, while proteins altered by DMBA in obese mice had 105 nodes and 120 edges **(**Supplemental Figure 1C**)**. In lean DMBA compared with obese DMBA mice, ovarian proteins altered had 514 nodes and 3688 edges (Supplemental Figure 1A**;**  *P* < 0.05).

### Obesity alters histones and histone variants in DMBA-exposed mice

Proteomic profiling of the ovary using LC–MS/MS determined that the abundance of histones and histone variants were altered in mice exposed to DMBA. In obese mice, basal levels of histone H3 fragment, histone H4, histone H1.2, histone H3.2, and histone H3.3 were decreased, whereas histone H1.1, histone H1.2, and histone H1.4 were increased as compared in lean mice **(**Table 1.1**;**  *P* < 0.1). In DMBA-exposed lean mice, retinoblastoma-binding protein 7 (RBBP7) was decreased compared with lean control mice **(**Table 1.2**;**  *P* < 0.1). In obese mice, exposure to DMBA increased the abundance of histone H2B **(**Table 1.3**;**  *P* < 0.1). Relative to lean DMBA exposed mice, DMBA-exposed obese mice had decreased the abundance of histone H4, histone H3.3, histone H3 fragment, core histone macro-H2A.1, histone domain-containing protein, core histone macro-H2A, and histone H2A type 1-F, whereas histone-binding protein RBBP7, histone H1.4, histone H1.1, histone 1.2, and H15 domain-containing protein were increased **(**Table 1.4**;**  *P* < 0.1). Some histone proteins were altered in more than one treatment comparison **(**Table 2**;**  *P* < 0.1). Similar abundance direction patterns were noted in all proteins presented in Table 2 except RBBP7, which was decreased in DMBA-exposed lean mice compared with the lean controls but increased due to obesity in the DMBA-exposed mice.

**Table 1.1:** List of histone proteins altered as a response to obesity (HPLC vs. HPOC).

Uniprot ID	Protein names	log2(FC)
A1L0V4	Histone H3 (fragment)	−5.98
P84228	Histone H3.2	−3.52
P62806	Histone H4	−3.22
P84244	Histone H3.3	−3.05
P15864	Histone H1.2	1.59
P43274	Histone H1.4	1.90
P43275	Histone H1.1	2.01
Q1WWK3	Hist1h1b protein (fragment)	2.14

**Table 1.2:** List of histone proteins altered as a response to DMBA (HPLC vs. HPLD).

Uniprot ID	Protein names	log2(FC)
Q60973	Histone-binding protein RBBP7	−0.57

**Table 1.3:** List of histone proteins altered as a response to DMBA in obese mice (HPOC vs. HPOD).

Uniprot ID	Protein names	log2(FC)
Q64525	Histone H2B type 2-B	0.31

**Table 1.4:** List of histone proteins altered as a response to obesity in DMBA-exposed mice (HPLD vs. HPOD).

Uniprot	Protein name	log2(FC)
P62806	Histone H4	−4.03
P84244	Histone H3.3	−3.71
A1L0V4	Histone H3 (fragment)	−3.45
Q9QZQ8	Core histone macro-H2A.1	−3.14
Q8C622	Histone domain-containing protein	−2.83
Q8BP16	Core histone macro-H2A	−2.19
Q8CGP5	Histone H2A type 1-F	−0.42
Q60973	Histone-binding protein RBBP7	0.99
P43274	Histone H1.4	1.45
P43275	Histone H1.1	2.09
P15864	Histone H1.2	2.72
Q3U292	H15 domain-containing protein	3.07

**Table 2:** Impact of DMBA on the total histone proteins profile in lean and obese mice ovaries.

Uniprot	Protein	HPLC versus HPOC log2(FC)	HPLC versus HPLD log2(FC)	HPOC versus HPOD log2(FC)	HPLD versus HPOD log2(FC)
A1L0V4	Histone H3 (fragment)	−5.9			−3.7
P62806	Histone H4	−3.2			−4
P84244	Histone H3.3	−3.1			−3.7
Q3U292	H15 domain-containing protein	2			3.1
P43275	Histone H1.1 (H1 VAR.3)	2			2.1
Q60973	Histone-binding protein RBBP7		−0.57		1
Q8VHL1	Histone-lysine N-methyltransferase SETD7			0.46	0.4

### Obesity decreases H3.3 in the ovary but does not alter its localization in follicles of DMBA-exposed mice

**Figure 3:**
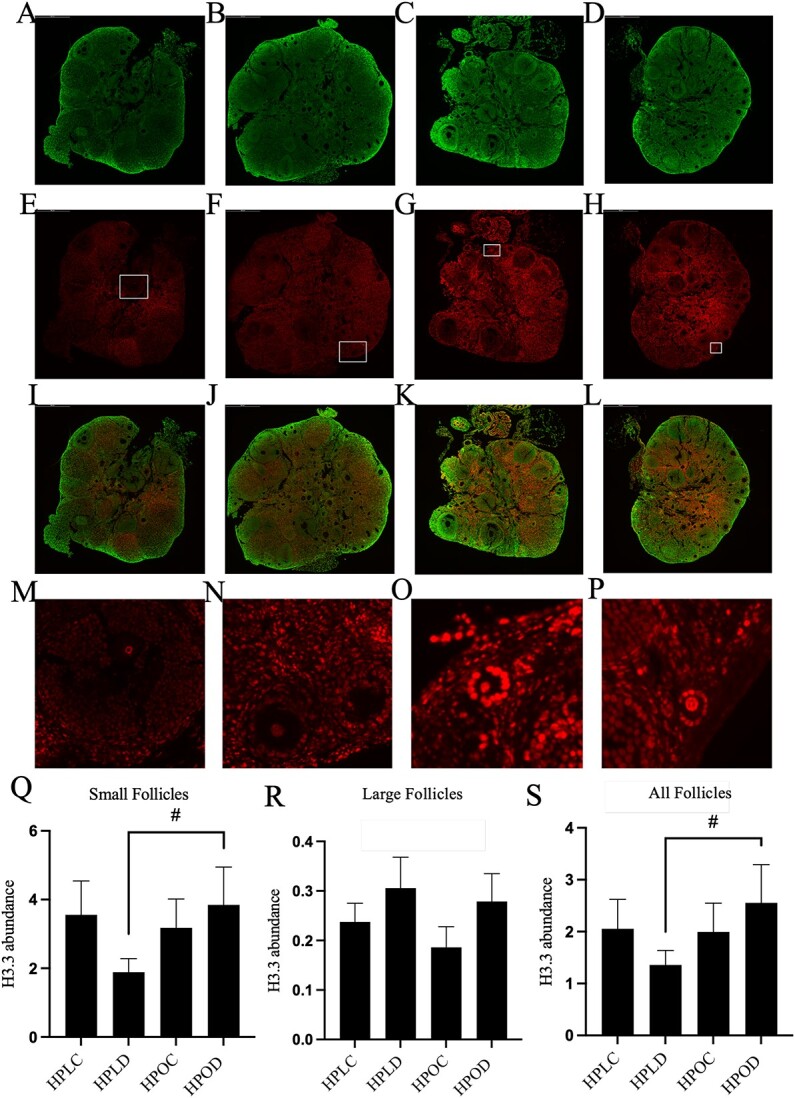
Impact of DMBA on ovarian H3.3 localization in lean and obese mice. Following 7 days of exposure to either vehicle control (CT) or DMBA, ovaries underwent immunostaining to detect H3.3 protein (red) in (A, E, I) HPLC; (B, F, J) HPLD; (C, G, K) HPOC; and (D, H, L) HPOD mouse ovaries. M–P: magnified follicles as indicted in the inserted boxes in E–H from each treatment. The intensity of H3.3 staining was quantified and statistically analyzed in (Q) small follicles, (R) large follicles, (S) all growing follicles. Cellular DNA is stained in green (A-D and I-L). Images were captured at 20x magnification. ^#^ indicates *P* < 0.1; *n* = 4.

The total ovarian abundance of the histone H3.3 was reduced in obese mice regardless of DMBA exposure **(**Table 2**)**. Thus, localization of H3.3 was analyzed by measuring the intensity of the stain in healthy follicles using ImageJ software. Histone H3.3 was identified as present in ovaries from all treatment groups (Figure 3A–L) and in all cell types in the ovary tissue. There was a tendency (*P* < 0.1) for greater H3.3 intensity in small follicles of ovaries of obese mice exposed to DMBA as compared with ovaries of lean mice exposed to DMBA (Figure 3Q**;**  *P* < 0.1). Two-way ANOVA analysis did not support an interaction effect (*P* = 0.18) between obesity and DMBA exposure on H3.3 abundance. A similar trend (*P* < 0.1) was observed when all follicle types were combined (Figure 3S; interaction effect: *P* = 0.27) and no changes (*P* > 0.05) to histone H3.3 were observed in the large follicles (interaction effect: *P* = 0.81; Figure 3R**)**. H3.3 protein staining was visualized in the oocyte nuclei of all developing follicle stages and was observably more intense in the oocyte nuclei of small follicles (Figure 3O and P) as compared with the oocyte nuclei of large follicles (Figure 3M and N).

## Discussion

The PAH group of chemicals are ubiquitous, and PAH exposure can induce dysfunction in the female reproductive system, including ovarian follicle destruction, impaired steroidogenesis, folliculogenesis, embryo transportation, and implantation and endometrial function [54–57]. The ovary expresses chemical biotransformation enzymes [58–61] which can bioactivate DMBA to a genotoxic and ovotoxic metabolite, 3,4-diol-1,2-epoxide [37]. The electrophilic property of this epoxide metabolite reacts with DNA, which acts as a nucleophile, leading to DNA adduct formation [29]. Ovotoxicity induction by DMBA has been documented in several species [37, 43–51]. In mice, DMBA exposure induces follicle loss in vivo [24] and ex vivo [62, 63], and DNA damage [26]. Neonatal rodent ovaries exposed to DMBA had alterations in signaling pathways involved in the regulation of follicular function at the messenger RNA (mRNA) and the protein levels [24, 64, 65]. Despite the use of DMBA as a model carcinogen and genotoxicant, how the ovaries respond to DMBA-induced DNA damage remains to be fully elucidated.

The ovarian response to environmental toxicants can be altered by obesity. Obesity alone compromises ovarian function by impairing folliculogenesis and steroidogenesis [66, 67], and inducing inflammation [68], DNA damage [26], and granulosa cell apoptosis [69]. We have previously noted that inflammatory markers are increased by obesity both in the ovary [66, 70] and in the surrounding peri-ovarian fat pad [70]. Additionally, basal ovarian DNA damage is apparent due to an obese phenotype [26]. Further, an impaired ovarian response to exposure to genotoxicants including zearalenone [25], cyclophosphamide [27], and DMBA [24, 26] has been reported, indicating a diminished capacity of the ovary to respond to chemical exposure in obese females. To respond to environmental genotoxic stress, histones undergo PTM or can sometimes be replaced by variant histones. Epigenetic alterations are prominent in ovarian cancer [71–73], although investigations into the roles of histones and histone modifications underlying ovarian pathology remain at a nascent. The epigenetic controls employed by the ovary to repair DNA damage resultant from environmental and chemotherapeutic exposures require better understanding. Thus, in this study, the impact of DMBA exposure on the ovarian proteomic response, changes in the ovarian histone profile, and the influence of obesity were interrogated. To do so, a mouse model of hyperphagic obesity was employed, to limit the confounding impact of diet composition. In order to avoid introducing variation due to stage of the estrous cycle, all ovaries were collected on Day 2 of diestrus with a maximum lag in tissue collection of 3 days post-cessation of DMBA exposure. This experimental paradigm was previously observed to have a basal impact of obesity on primary, preantral, and corpora lutea numbers which were reduced [53]. A differential response to DMBA was also reported with a tendency for a reduced primordial follicle number in lean mice and an increased primary follicle number in obese mice as a result of DMBA exposure [53]. There was also evidence of DNA damage induction due to DMBA exposure, with both phosphorylated histone 2AX (γH2AX) and breast cancer 1 (BRCA1) classic DNA repair proteins being observed albeit decreased in some follicle stages by DMBA exposure [53]. Thus, this model is suitable to allow interrogation of initiating ovotoxic mechanisms in the absence of overt follicle destruction.

Exposure to DMBA initiated an ovarian proteomic response in both lean and obese mice, although it was more pronounced in obese mice; 120 proteins were altered in obese mice exposed to DMBA as compared with 48 in the lean mice who were exposed identically. Notably, the proteomic response in lean and obese mice was largely heterogenous, although some overlap was observed when the *P*-value cutoff was relaxed to *P* < 0.1. Four overlapping proteins, proteasome subunit beta type-3 (PSMB3), serotransferrin (TF), small monomeric GTPase (GM9392), and disabled homolog 2 (DAB2) proteins, were observed to be altered in every treatment comparison. During DMBA exposure, a more dramatic response was noted in the obese relative to the lean exposed mice, suggesting either that the amount of ovarian exposure differed due to systemic impacts (DMBA being a lipophilic molecule) and impairment in ovarian DMBA detoxification, or due to an impaired ability to execute an effective DDR response. In obese relative to lean mice exposed to DMBA, epoxide hydrolase 2 (EPHX2) was a notable protein that was decreased due to DMBA exposure. It is known that EPHX1 bioactivates DMBA [61] and elevated EPHX1 mRNA and protein levels are observed in 18-week-old obese relative to lean mice [24] but EPHX2 is known to be involved in the metabolism of epoxides derived from fatty acids [74] and has not been previously investigated in terms of DMBA biotransformation. The absence of changes to ovarian EPHX2 in lean mice may suggest a differential DMBA biotransformation response due to metabolic status.

Untargeted proteomic profiling provided insight into the general classes of proteins altered by DMBA exposure in lean and obese mice, generating patterns of the cellular responses initiated. Since an aim of this study was to assess the ovarian DDR and to identify if alterations to histones occurred due to DMBA exposure, impacts on histone proteins were evaluated. Histone proteins are generally under-investigated in ovotoxicity because the mRNA expression changes in histones may often go unnoticed due to their exclusions from microarray profiling [75]. Despite our previous observations that DMBA-exposed lean and obese mice did not have modifications to ovarian H3K4me, H4K5ac, H4K12ac, and H4K16ac [53] which have been associated with the DDR. In this study histones H3 and H4 were reduced in both lean and obese mice exposed to DMBA indicating that DMBA may target histones as a mechanism of ovotoxicity. The tails of histones H3 and H4 provide sites for epigenetic modification and changes in their distributions can subsequently lead to changes to the epigenome. The presence of DNA hypomethylation in tumor cells has been established [76–78] potentially leading to genomic instability. Genome-wide hypomethylation is also observed in ovarian cancers [79] and exposure to environmental toxicants and obesity could plausibly alter methylation patterns by modifying histone protein distribution, as observed in this study. A slight increase in the abundance of histone H2b was noted in obese mice exposed to DMBA as compared with obese control mice. Ubiquitylation of H2b contributes to bypassing DNA damage which is important for the defense against genome instability, mutations, and cancer [68]. Obese mouse ovaries, regardless of DMBA exposure, had increased the abundance of histone 1 variants, which, in general, mediate transcriptional repression. Progressing ovarian tumors have been associated with a reduction in the mRNA of somatic histone 1 variants [75] and herein we observed an increase in the abundance of somatic histone variants, i.e., H1.1, H1.2, and H1.4, suggestive of a molecular response potentially preceding an adverse phenotype. Thus, alterations to histone proteins and variants due to DMBA exposure and/or obesity were noted, potentially indicating their roles in the ovarian response to environmental and systemic stress.

Histone proteins that were altered in more than one treatment comparison mainly had similar patterns of change, with the exception of RBBP7. Loss of RBBP7 is associated with changes to heterochromatin structure followed by accumulation of DNA damage [80] and RBBP7 is also a component of epigenetic complexes which regulate transcription [81], and interestingly, it also has a role in H3.3 regulation [81]. An RBBP7 reduction in ovaries from DMBA-exposed lean mice was observed compared with their respective controls, but the abundance of RBBP7 was increased in obese mice exposed to DMBA when compared with identically exposed lean mice.

It is important to note that in contrast to our expectations, DMBA exposure had fewer effects on the histone profile than obesity alone. One of the interesting histone variants which was reduced altered by obesity was histone H3.3, a constitutively expressed form of H3, which is required for transcriptional memory and primes genes for activation after genotoxic stress [82, 83]. The observed H3.3 reduction in obese mouse ovaries could indicate a lag in activation of the ovarian DDR. The involvement of H3.3 chromatin dynamics post-DNA damage [84] suggested that it was an interesting candidate protein to study in the context of ovarian genotoxicant exposure, although there is scant information about the basal ovarian functionality. To identify the ovarian localization of H3.3, immunostaining was employed and H3.3 was observed to be ubiquitous in the ovary and present in all treatment group ovaries. H3.3 staining was also observed in the oocyte nuclei of all follicles including primary, secondary, and antral follicles, although the intensity of the stain was visibly higher in the oocyte nuclei of small follicles. In small pre-antral follicles of DMBA-exposed obese mice, the H3.3 staining intensity tended to increase, which could indicate a delayed response to the genotoxic exposure or heightened DNA damage. In larger follicles, H3.3 abundance, however, remained unchanged, thus the response is likely to differ due to DMBA exposure not only in lean and obese mice but also between specific follicular development stages.

Although the stage of the estrous cycle was deemed more important at the time of euthanasia to control for any confounding effects that may occur due to the hormonal profile, some discrepancies in the proteomic response may exist due to the short time elapsed between cessation of exposure and euthanasia so the immediate DDR may have ceased, and value could be gained by examining the immediate effects of DMBA exposure. It is also to be noted that histones are synthesized in the cytoplasm, transported to the nucleus, and actively incorporated into newly replicated DNA [85]. Thus, the histone profile represented in this proteome may mostly be representative of the newly synthesized histones and exclusive of the chromatin-incorporated histones, thus isolating the analysis to nuclear proteins would be of value. Despite these limitations, a differentially abundant histone profile due to genotoxicant exposure was observed and the response differed between ovaries of lean and obese females, opening avenues to explore the importance of potential epigenetic alterations in obese individuals and their impact post-ovotoxicant exposure(s). It should be noted that the alterations to follicle number observed in the phenotypic and morphological outcomes of this experimental paradigm could have affected the proteins reported in this study, albeit this would be a secondary effect of DMBA exposure and/or obesity, but is worthy of consideration.

In summary, exposure to DMBA initiates an ovarian proteomic response which differs between lean and obese mice as noted in previous studies with other ovotoxicants [24, 25, 86]. A differential impact on the distribution of ovarian histones and histone variants in lean and obese mice exposed to DMBA was observed. Although the overall level of histone H3.3 was reduced in obese mice, a higher staining intensity for H3.3 in oocytes of small pre-antral follicles in DMBA-exposed obese mice was noted. Generally, post-translationally active histones H3 and H4 were decreased, whereas histone H1, which is involved in transcription repression, was increased in obese but not in lean mice. Taken together, these data suggest a role for alterations to histones during the ovarian DDR and that obesity alters the distribution of individual histone proteins, potentially altering transcriptional regulation both basally and in the ovaries of DMBA-exposed mice.

## Conflict of Interest

The authors have declared that no conflict of interest exists.

## Data availability

Data are available upon request.

## Authors’ contributions

AFK contributed to experimental conception and design. KT and HEW performed animal study and tissue collection. KCK provided intellectual and technical assistance with microscopy. JKR performed the experiments and data analysis. JKR drafted the manuscript. AFK reviewed, edited, and approved the final manuscript.

## Supplementary Material

supplemental_table_1_ioad140

supplemental_table_2_ioad140

supplemental_table_3_ioad140

supplemental_table_4_ioad140

supplemental_table_5_ioad140

supplemental_table_6_ioad140

supplemental_table_7_100623_ioad140

supplemental_figure_1_ioad140
