# Supplementary material for: Altered histone abundance as a mode of ovotoxicity during 7,12-dimethylbenz[a]anthracene exposure with additive influence of obesity
Source: Biol Reprod. 2023 Oct 19;110(2):419–29. doi: 10.1093/biolre/ioad140 (PMC10873273; doi:10.1093/biolre/ioad140)
Supplement: supplemental_table_1_ioad140 [file supplemental_table_1_ioad140.docx]

**Supplemental Table 1.**  **Impact of obesity on the ovarian protein abundance.** After 7 d of exposure to vehicle control or DMBA in lean and obese mice, total ovarian proteome changes were quantified via LC-MS/MS. All proteins (225) altered by obesity are listed (*P* < 0.05; n = 5).

| **Uniprot ID** | **Protein names** | **Log2(FC)** | **q-value** |
| --- | --- | --- | --- |
| Q8R0F8 | Acylpyruvase FAHD1 | -6.75 | < 0.05 |
| A1L0V4 | Histone H3 (Fragment) | -5.98 | 0.117 |
| P00493 | Hypoxanthine-guanine phosphoribosyltransferase | -5.76 | < 0.05 |
| Q99L27 | GMP reductase 2 | -5.61 | 0.189 |
| O35490 | Betaine--homocysteine S-methyltransferase 1 | -5.58 | 0.236 |
| B1AU71 | DBF4-type zinc finger-containing protein 2 homolog (Fragment) | -5.36 | 0.174 |
| Q9R1T2 | SUMO-activating enzyme subunit 1 | -4.91 | 0.073 |
| O08738 | Caspase-6 | -3.95 | 0.268 |
| Q9DBB8 | Trans-1,2-dihydrobenzene-1,2-diol dehydrogenase | -3.90 | 0.195 |
| Q8JZM0 | Dimethyladenosine transferase 1 | -3.62 | 0.067 |
| P84228 | Histone H3.2 | -3.52 | 0.083 |
| P70677 | Caspase-3 | -3.42 | < 0.05 |
| P17563 | Methanethiol oxidase | -3.35 | < 0.05 |
| Q5D098 | Proteasome subunit beta (EC 3.4.25.1) (Fragment) | -3.33 | 0.105 |
| P62806 | Histone H4 | -3.22 | 0.079 |
| Q9JHU9 | Inositol-3-phosphate synthase 1 | -3.22 | 0.059 |
| O08749 | Dihydrolipoyl dehydrogenase | -3.18 | 0.123 |
| P46412 | Glutathione peroxidase 3 | -3.18 | 0.171 |
| D3YTP8 | U6 snRNA-associated Sm-like protein LSm4 (Fragment) | -3.17 | < 0.05 |
| Q9D2G2 | Dihydrolipoyllysine-residue succinyltransferase component of 2-oxoglutarate dehydrogenase complex | -3.05 | 0.172 |
| P84244 | Histone H3.3 | -3.05 | 0.065 |
| Q3UGR5 | Haloacid dehalogenase-like hydrolase domain-containing protein 2 | -3.04 | 0.259 |
| Q9DCT2 | NADH dehydrogenase [ubiquinone] iron-sulfur protein 3 | -3.03 | 0.070 |
| U5LP42 | Anti-H5N1 hemagglutinin monoclonal anitbody H5M9 heavy chain (Fragment) | -2.90 | 0.111 |
| O35900 | U6 snRNA-associated Sm-like protein LSm2 (Protein G7b) | -2.89 | 0.056 |
| A0A0R4J131 | Biotinidase | -2.86 | < 0.05 |
| Q8C8Z0 | Uncharacterized protein (Fragment) | -2.83 | 0.271 |
| Q71RI9 | Kynurenine--oxoglutarate transaminase 3 | -2.70 | 0.093 |
| P62313 | U6 snRNA-associated Sm-like protein LSm6 | -2.68 | 0.065 |
| F6TB64 | Ubinuclein-2 (Fragment) | -2.62 | 0.212 |
| Q9D819 | Inorganic pyrophosphatase (EC 3.6.1.1) | -2.51 | 0.202 |
| Q9D172 | Glutamine amidotransferase-like class 1 domain-containing protein 3A | -2.43 | 0.201 |
| Q3U3C2 | Epididymal secretory protein E1 (NPC intracellular cholesterol transporter 2) | -2.41 | 0.115 |
| P56480 | ATP synthase subunit beta, mitochondrial (EC 7.1.2.2) (ATP synthase F1 subunit beta) | -2.36 | 0.179 |
| P00329 | Alcohol dehydrogenase 1 (EC 1.1.1.1) (ADH-A2) (Alcohol dehydrogenase A subunit) | -2.34 | 0.144 |
| G3X8Q5 | Ceruloplasmin | -2.33 | 0.066 |
| D3Z5P0 | Serine/threonine-protein kinase BRSK1 | -2.31 | 0.084 |
| P62307 | Small nuclear ribonucleoprotein F (snRNP-F) (Sm protein F) (Sm-F) (SmF) | -2.28 | 0.063 |
| Q9Z2U1 | Proteasome subunit alpha type-5 | -2.27 | 0.253 |
| P20108 | Thioredoxin-dependent peroxide reductase | -2.26 | 0.207 |
| Q9CPY7 | Cytosol aminopeptidase | -2.22 | 0.277 |
| Q62377 | U2 small nuclear ribonucleoprotein auxiliary factor 35 kDa subunit-related protein 2 | -2.12 | 0.106 |
| A0A0R4J138 | Arylsulfatase B | -2.09 | 0.279 |
| P97807 | Fumarate hydratase | -2.05 | 0.179 |
| Q9Z1Z2 | Serine-threonine kinase receptor-associated protein (UNR-interacting protein) | -2.01 | 0.123 |
| Q8C2S9 | Major vault protein | -1.93 | 0.203 |
| Q3U8H8 | NTF2 domain-containing protein | -1.86 | 0.062 |
| P09671 | Superoxide dismutase [Mn] | -1.85 | < 0.05 |
| B1AWZ5 | Protein NipSnap homolog 3B | -1.85 | 0.254 |
| O09061 | Proteasome subunit beta type-1 | -1.84 | 0.187 |
| Q9WTX5 | S-phase kinase-associated protein 1 | -1.83 | 0.164 |
| Q9R1P1 | Proteasome subunit beta type-3 | -1.78 | 0.066 |
| O70433 | Four and a half LIM domains protein 2 | -1.75 | 0.056 |
| O70423 | Membrane primary amine oxidase | -1.71 | 0.281 |
| P14069 | Protein S100-A6 (5B10) (Calcyclin) | -1.69 | 0.068 |
| Q5DU67 | MFLJ00088 protein (Fragment) | -1.65 | 0.259 |
| P10493 | Nidogen-1 (NID-1) (Entactin) | -1.63 | 0.066 |
| Q921I1 | Serotransferrin (Transferrin) | -1.63 | < 0.05 |
| Q3U1J4 | DNA damage-binding protein 1 (DDB p127 subunit) | -1.62 | 0.121 |
| F8WIV2 | Serine (or cysteine) peptidase inhibitor | -1.62 | 0.066 |
| C5H0E8 | Rap1A-retro1 | -1.60 | 0.060 |
| G5E8I8 | Calcium homeostasis endoplasmic reticulum protein | -1.58 | 0.177 |
| E9Q2S9 | Coiled-coil domain-containing protein 43 | -1.57 | 0.121 |
| Q921M3 | Splicing factor 3B subunit 3 | -1.51 | 0.238 |
| Q61171 | Peroxiredoxin-2 | -1.46 | 0.207 |
| P97315 | Cysteine and glycine-rich protein 1 | -1.45 | 0.234 |
| Q9CQJ6 | Density-regulated protein (DRP) | -1.45 | 0.068 |
| Q08879 | Fibulin-1 (FIBL-1) | -1.45 | 0.065 |
| Q80YX1 | Tenascin (TN) (Hexabrachion) | -1.39 | 0.222 |
| Q9D6J6 | NADH dehydrogenase [ubiquinone] flavoprotein 2 | -1.37 | 0.068 |
| P62259 | 14-3-3 protein epsilon (14-3-3E) | -1.36 | 0.110 |
| Q60692 | Proteasome subunit beta type-6 | -1.35 | 0.118 |
| P61358 | 60S ribosomal protein L27 | -1.35 | 0.273 |
| Q6PCP0 | RNA helicase | -1.32 | 0.241 |
| P21981 | Protein-glutamine gamma-glutamyltransferase 2 | -1.30 | 0.175 |
| Q8VDM6 | Heterogeneous nuclear ribonucleoprotein U-like protein 1 | -1.30 | 0.117 |
| Q8R3Q6 | Coiled-coil domain-containing protein 58 | -1.28 | 0.098 |
| P63101 | 14-3-3 protein zeta/delta | -1.28 | 0.253 |
| Q7TMQ1 | Gap junction protein | -1.27 | 0.258 |
| Q9CX80 | Cytoglobin (Histoglobin) | -1.22 | 0.189 |
| P01027 | Complement C3 (HSE-MSF) | -1.22 | 0.276 |
| P21614 | Vitamin D-binding protein (DBP) | -1.19 | 0.068 |
| P35700 | Peroxiredoxin-1 | -1.13 | 0.099 |
| Q9CQF7 | Prefoldin 1 | -1.09 | < 0.05 |
| Q3UWG5 | Tetraspanin | -1.07 | 0.215 |
| P61982 | 14-3-3 protein gamma | -1.02 | 0.254 |
| P63168 | Dynein light chain 1 | -1.01 | 0.089 |
| Q9QUH0 | Glutaredoxin-1 (Thioltransferase-1) | -0.96 | 0.087 |
| Q52KG9 | Chaperonin containing Tcp1, subunit 6a (Zeta) | -0.93 | 0.268 |
| Q61233 | Plastin-2 (65 kDa macrophage protein) | -0.90 | 0.127 |
| Q9CPU0 | Lactoylglutathione lyase | -0.90 | 0.181 |
| D3Z4U0 | Zinc finger Ran-binding domain-containing protein 2 | -0.84 | 0.111 |
| Q8CFZ6 | C-type lectin domain family 3 | -0.70 | 0.200 |
| B1B0C7 | Basement membrane-specific heparan sulfate proteoglycan core protein | -0.62 | 0.116 |
| P47911 | 60S ribosomal protein L6 | -0.44 | 0.265 |
| P70296 | Phosphatidylethanolamine-binding protein 1 | 0.32 | 0.250 |
| Q569Z6 | Thyroid hormone receptor-associated protein 3 | 0.32 | 0.277 |
| Q3UVN5 | NSFL1 cofactor p47 | 0.35 | 0.163 |
| P57776 | Elongation factor 1-delta (EF-1-delta) | 0.37 | 0.070 |
| Q9JKR6 | Hypoxia up-regulated protein 1 | 0.37 | 0.096 |
| P14211 | Calreticulin (CRP55) | 0.39 | 0.201 |
| P18760 | Cofilin-1 (Cofilin, non-muscle isoform) | 0.41 | 0.233 |
| Q8BQ02 | Uncharacterized protein | 0.45 | 0.187 |
| Q9DBR0 | A-kinase anchor protein 8 | 0.47 | 0.219 |
| P62869 | Elongin-B (EloB) | 0.47 | 0.163 |
| F6ZFU0 | Elongation factor 1-delta (Fragment) | 0.49 | 0.279 |
| P57759 | Endoplasmic reticulum resident protein 29 (ERp29) | 0.50 | 0.284 |
| G5E8R8 | UBX domain-containing protein 7 | 0.53 | 0.266 |
| Q6XLQ8 | Calumenin | 0.53 | 0.119 |
| F8VQJ3 | Laminin subunit gamma-1 | 0.54 | 0.066 |
| P63028 | Translationally-controlled tumor protein | 0.55 | 0.278 |
| P48024 | Eukaryotic translation initiation factor 1 | 0.56 | 0.086 |
| Q8C1W9 | Uncharacterized protein | 0.57 | 0.118 |
| Q3UXU0 | Uncharacterized protein | 0.58 | 0.098 |
| Q8CCS6 | Polyadenylate-binding protein 2 | 0.58 | 0.056 |
| Q8CJ40 | Rootletin (Ciliary rootlet coiled-coil protein) | 0.58 | 0.261 |
| P38647 | Stress-70 protein | 0.58 | 0.177 |
| Q6R891 | Neurabin-2 | 0.59 | 0.261 |
| P98078 | Disabled homolog 2 | 0.60 | 0.279 |
| Q91YI0 | Argininosuccinate lyase | 0.61 | 0.146 |
| O70456 | 14-3-3 protein sigma (Stratifin) | 0.61 | 0.223 |
| A0A0G2JFX7 | RNA-binding protein 8A | 0.61 | 0.213 |
| Q9CQH7 | Transcription factor BTF3 homolog 4 | 0.62 | 0.213 |
| Q9D281 | Protein Noxp20 | 0.62 | 0.264 |
| Q3UF30 | Calpactin I light chain | 0.62 | 0.178 |
| Q9CR51 | V-type proton ATPase subunit G 1 | 0.63 | 0.231 |
| Q3TWW8 | Serine/arginine-rich splicing factor 6 | 0.63 | 0.189 |
| P54071 | Isocitrate dehydrogenase [NADP] | 0.63 | 0.252 |
| Q9CPR4 | 60S ribosomal protein L17 | 0.65 | 0.202 |
| Q91WG2 | Rab GTPase-binding effector protein 2 (Rabaptin-5beta) | 0.66 | 0.188 |
| Q19LI2 | Alpha-1B-glycoprotein | 0.69 | 0.071 |
| Q80U76 | Ribosome biogenesis regulatory protein (Fragment) | 0.69 | 0.227 |
| E9PV24 | Fibrinogen alpha chain | 0.70 | 0.265 |
| P99027 | 60S acidic ribosomal protein P2 | 0.70 | 0.115 |
| P49817 | Caveolin-1 | 0.71 | 0.198 |
| P70297 | Signal transducing adapter molecule 1 (STAM-1) | 0.72 | 0.264 |
| Q9D0B6 | Protein PBDC1 (Polysaccharide biosynthesis domain-containing protein 1) | 0.72 | 0.198 |
| P16546 | Spectrin alpha chain | 0.72 | 0.068 |
| Q3UJR8 | Transcription factor BTF3 | 0.73 | 0.209 |
| F6RDI8 | Serine and arginine-rich-splicing factor 11 (Fragment) | 0.73 | 0.066 |
| P62830 | 60S ribosomal protein L23 | 0.73 | 0.068 |
| Q00899 | Transcriptional repressor protein YY1 | 0.74 | 0.117 |
| Q3UAI4 | SAP domain-containing protein | 0.74 | 0.262 |
| P27773 | Protein disulfide-isomerase A3 | 0.76 | 0.080 |
| P46664 | Adenylosuccinate synthetase isozyme 2 | 0.77 | 0.281 |
| Q99J29 | Carboxypeptidase | 0.78 | 0.120 |
| Q61990 | Poly(rC)-binding protein 2 | 0.79 | 0.239 |
| P20029 | Endoplasmic reticulum chaperone BiP | 0.79 | < 0.05 |
| A0A0R4J0Q5 | Lamin-B2 | 0.80 | 0.218 |
| Q62376 | U1 small nuclear ribonucleoprotein 70 kDa | 0.80 | 0.085 |
| P10852 | 4F2 cell-surface antigen heavy chain | 0.82 | 0.068 |
| Q70IV5 | Synemin (Desmuslin) | 0.82 | 0.174 |
| Q6NZB0 | DnaJ homolog subfamily C member 8 | 0.82 | 0.187 |
| Q9Z0F7 | Gamma-synuclein (Persyn) | 0.83 | 0.281 |
| Q5M9N5 | 60S ribosomal protein L28 | 0.84 | 0.266 |
| Q69ZX3 | MKIAA0866 protein (Fragment) | 0.84 | 0.201 |
| Q9DBB9 | Carboxypeptidase N subunit 2 | 0.86 | 0.200 |
| Q3UMT1 | Protein phosphatase 1 regulatory subunit 12C | 0.86 | 0.223 |
| E9Q447 | Spectrin alpha chain, non-erythrocytic 1 | 0.87 | 0.146 |
| Q9Z1D1 | Eukaryotic translation initiation factor 3 subunit G | 0.88 | 0.063 |
| Q3TDX7 | Extracellular matrix protein 1 | 0.91 | < 0.05 |
| Q641N3 | Rps16 protein (Fragment) | 0.92 | 0.201 |
| Q64105 | Sepiapterin reductase (SPR) | 0.93 | 0.267 |
| Q3U6S1 | Vimentin | 0.94 | 0.190 |
| T1ECW4 | RNA-binding protein with multiple-splicing | 0.94 | 0.068 |
| O35685 | Nuclear migration protein nudC | 0.95 | < 0.05 |
| Q91WN1 | DnaJ homolog subfamily C member 9 | 0.97 | 0.093 |
| Q9CPX4 | Ferritin | 0.98 | 0.267 |
| Q8K144 | Polypyrimidine tract-binding protein 1 | 0.98 | 0.071 |
| Q62261 | Spectrin beta chain, non-erythrocytic 1 (Beta-II spectrin) | 0.98 | 0.200 |
| P06728 | Apolipoprotein A-IV | 0.99 | 0.200 |
| A0A140LID0 | Probable global transcription activator SNF2L2 (Fragment) | 1.01 | 0.106 |
| Q80TM2 | MKIAA1027 protein (Fragment) | 1.03 | 0.060 |
| P47955 | 60S acidic ribosomal protein P1 | 1.04 | 0.272 |
| Q3TIH8 | Uncharacterized protein | 1.05 | 0.119 |
| Q6NZD2 | Sorting nexin-1 | 1.06 | 0.162 |
| Q8R081 | Heterogeneous nuclear ribonucleoprotein L (hnRNP L) | 1.08 | 0.119 |
| P60840 | Alpha-endosulfine (ARPP-19e) | 1.09 | 0.189 |
| Q9DCV7 | Keratin, type II cytoskeletal 7 | 1.10 | 0.167 |
| A0A2I3BPG9 | Ribosomal protein L36A, pseudogene 1 | 1.11 | 0.149 |
| O88207 | Collagen alpha-1(V) chain | 1.11 | 0.259 |
| Q3V2C6 | IF rod domain-containing protein | 1.14 | 0.179 |
| Q6LD55 | Apolipoprotein A-II (Apolipoprotein A2) | 1.17 | 0.115 |
| Q8BSH9 | Nucleosome assembly protein 1-like 1 | 1.21 | < 0.05 |
| Q8BMK4 | Cytoskeleton-associated protein 4 | 1.21 | 0.100 |
| P62918 | 60S ribosomal protein L8 | 1.21 | 0.061 |
| A2AEW9 | GRIP1-associated protein 1 | 1.21 | 0.189 |
| Q6GTX3 | Apoe protein (Apolipoprotein E) | 1.22 | 0.070 |
| Q99K41 | EMILIN-1 (Elastin microfibril interface-located protein 1) | 1.24 | 0.129 |
| P41317 | Mannose-binding protein C | 1.24 | 0.282 |
| Q3UKW1 | SERPIN domain-containing protein | 1.25 | 0.117 |
| A0A1L7NR37 | Arg/Abl-binding protein 2 | 1.26 | 0.062 |
| B1ARU4 | Microtubule-actin cross-linking factor 1 | 1.26 | 0.143 |
| P09528 | Ferritin heavy chain (Ferritin H subunit) | 1.27 | 0.069 |
| Q3UTI7 | Peptidyl-prolyl cis-trans isomerase | 1.27 | 0.220 |
| G3X8T2 | Zinc finger CCCH domain-containing protein 18 | 1.28 | 0.109 |
| Q3UW40 | TRASH domain-containing protein | 1.29 | 0.166 |
| P19001 | Keratin, type I cytoskeletal 19 | 1.30 | 0.096 |
| H7BX64 | Sarcolemmal membrane-associated protein | 1.32 | 0.147 |
| E9QJT5 | Acylphosphatase | 1.36 | 0.179 |
| P47963 | 60S ribosomal protein L13 (A52) | 1.36 | 0.066 |
| P62900 | 60S ribosomal protein L31 | 1.36 | 0.114 |
| O54724 | Caveolae-associated protein 1 (Cav-p60) | 1.38 | < 0.05 |
| P28654 | Decorin (Bone proteoglycan II) | 1.42 | 0.084 |
| Q3UYZ8 | Uncharacterized protein (Fragment) | 1.47 | 0.265 |
| O54941 | SWI/SNF-related matrix-associated actin-dependent regulator of chromatin subfamily E member 1 | 1.52 | 0.270 |
| P28653 | Biglycan (Bone/cartilage proteoglycan I) | 1.55 | 0.066 |
| Q3U4Y0 | H15 domain-containing protein | 1.59 | 0.059 |
| P15864 | Histone H1.2 | 1.59 | 0.116 |
| P07309 | Transthyretin (Prealbumin) | 1.61 | 0.098 |
| P11679 | Keratin, type II cytoskeletal 8 | 1.66 | 0.189 |
| Q01149 | Collagen alpha-2(I) chain | 1.70 | 0.255 |
| Q5FWB6 | 60S acidic ribosomal protein P0 | 1.72 | 0.069 |
| P05784 | Keratin, type I cytoskeletal 18 | 1.83 | 0.180 |
| Q545F8 | 40S ribosomal protein S4 | 1.86 | 0.188 |
| Q80YP5 | Integrin alpha 5 (Fibronectin receptor alpha) | 1.90 | 0.069 |
| P43274 | Histone H1.4 (H1 VAR.2) | 1.90 | 0.068 |
| Q3U292 | H15 domain-containing protein | 2.01 | 0.097 |
| P43275 | Histone H1.1 (H1 VAR.3) | 2.01 | 0.098 |
| Q1WWK3 | Hist1h1b protein (Fragment) | 2.14 | 0.070 |
| Q91XL1 | Leucine-rich HEV glycoprotein | 2.58 | 0.065 |
| Q8K411 | Presequence protease | 2.62 | 0.223 |
| A0A1B0GSF9 | Predicted gene, 19935 | 3.95 | 0.180 |
| Q8CC13 | AP complex subunit beta | 5.19 | 0.070 |
| P12658 | Calbindin (Calbindin D28) | 5.47 | 0.202 |
