# Supplementary material for: Altered histone abundance as a mode of ovotoxicity during 7,12-dimethylbenz[a]anthracene exposure with additive influence of obesity
Source: Biol Reprod. 2023 Oct 19;110(2):419–29. doi: 10.1093/biolre/ioad140 (PMC10873273; doi:10.1093/biolre/ioad140)
Supplement: supplemental_table_2_ioad140 [file supplemental_table_2_ioad140.docx]

**Supplemental Table 2.** Proteins affected by obesity involved in reproductive and DNA repair pathways (*P* < 0.05; n = 5).

| **Pathway** | **# of gene hits** | **Gene Name** | **Uniprot ID** |
| --- | --- | --- | --- |
| DNA replication (P00017) | 3 | Histone H3.2 | P84228 |
|  |  | Histone H3.3 | P84244 |
|  |  | Proliferating cell nuclear antigen | P17918 |
| Gonadotropin-releasing hormone receptor pathway (P06664) | 2 | Caveolin-1 | P49817 |
|  |  | Astrocytic phosphoprotein PEA-15 | Q62048 |
| PI3 kinase pathway (P00048) | 1 | 14-3-3 protein zeta/delta | P63101 |
| p53 pathway (P00059) | 1 | 14-3-3 protein sigma | O70456 |
