# Supplementary material for: Altered histone abundance as a mode of ovotoxicity during 7,12-dimethylbenz[a]anthracene exposure with additive influence of obesity
Source: Biol Reprod. 2023 Oct 19;110(2):419–29. doi: 10.1093/biolre/ioad140 (PMC10873273; doi:10.1093/biolre/ioad140)
Supplement: supplemental_table_3_ioad140 [file supplemental_table_3_ioad140.docx]

**Supplemental Table 3.**  **Impact of DMBA exposure on ovarian protein abundance in lean mice.** After 7 d of exposure to vehicle control or DMBA in lean and obese mice, total ovarian proteome changes were quantified via LC-MS/MS. Proteins (48) altered (*P* < 0.05; n = 5) by DMBA in lean mice are listed.

| **Uniprot ID** | **Protein names** | **Log2(FC)** | **q-value** |
| --- | --- | --- | --- |
| Q3UW40 | TRASH domain-containing protein | -1.76 | 1.31 |
| Q91Z53 | Glyoxylate reductase/hydroxypyruvate reductase | -1.75 | 1.36 |
| F2Z455 | Four and a half LIM domains protein 3 | -1.70 | 1.62 |
| Q8VCG1 | Deoxyuridine 5'-triphosphate nucleotidohydrolase (dUTPase) | -1.56 | 1.22 |
| Q99NB8 | Ubiquilin-4 (Ataxin-1 interacting ubiquitin-like protein) | -1.43 | 1.28 |
| E0CYH0 | Female-lethal(2)D homolog | -1.31 | 2.17 |
| Q99LP6 | GrpE protein homolog 1 | -1.29 | 1.21 |
| Q9QZM0 | Ubiquilin-2 (Chap1) | -1.24 | 1.95 |
| Q9DCG9 | Multifunctional methyltransferase subunit TRM112-like protein | -1.23 | 1.45 |
| A0A3B2WDD2 | Ribosomal protein | -1.16 | 1.26 |
| Q9CZM2 | 60S ribosomal protein L15 | -1.12 | 1.34 |
| Q9CQR2 | 40S ribosomal protein S21 | -1.10 | 1.30 |
| P47963 | 60S ribosomal protein L13 (A52) | -1.05 | 1.29 |
| Q3UXW3 | RNA helicase | -0.94 | 1.31 |
| P68040 | Receptor of activated protein C kinase 1 | -0.89 | 1.30 |
| A0A1L1SSH9 | Osteonectin (SPARC) | -0.88 | 1.53 |
| A2AVR9 | Dynein light chain roadblock | -0.87 | 1.40 |
| P62830 | 60S ribosomal protein L23 | -0.84 | 1.47 |
| Q80SZ7 | Guanine nucleotide-binding protein G(I)/G(S)/G(O) subunit gamma-5 | -0.81 | 1.39 |
| Q62376 | U1 small nuclear ribonucleoprotein 70 kDa | -0.76 | 1.22 |
| P47911 | 60S ribosomal protein L6 | -0.74 | 2.47 |
| T1ECW4 | RNA-binding protein with multiple-splicing | -0.70 | 1.46 |
| P10852 | 4F2 cell-surface antigen heavy chain | -0.70 | 1.23 |
| Q3TE63 | Peptidyl-prolyl cis-trans isomerase | -0.68 | 1.37 |
| Q9EST5 | Acidic leucine-rich nuclear phosphoprotein 32 family member B | -0.64 | 1.28 |
| G5E8V8 | Phosphorylated adapter RNA export protein | -0.58 | 1.07 |
| Q9JKR6 | Hypoxia up-regulated protein 1 (GRP-170) | -0.57 | 1.25 |
| P62852 | 40S ribosomal protein S25 | -0.50 | 1.38 |
| Q3TIQ2 | 60S ribosomal protein L12 | -0.47 | 1.38 |
| A0A0R4J039 | Histidine-rich glycoprotein | 0.28 | 1.27 |
| Q921I1 | Serotransferrin (Transferrin) | 0.44 | 1.37 |
| Q9DCC5 | Cbx3 protein (Chromobox protein homolog 3) | 0.51 | 1.42 |
| E9Q5L2 | Inter alpha-trypsin inhibitor | 0.56 | 1.30 |
| Q9CQF7 | Prefoldin 1 (Prefoldin subunit 1) | 0.70 | 1.12 |
| O35900 | U6 snRNA-associated Sm-like protein LSm2 (Protein G7b) | 0.71 | 1.34 |
| P62307 | Small nuclear ribonucleoprotein F (snRNP-F) | 0.73 | 1.18 |
| Q8CFZ6 | C-type lectin domain family 3 | 0.78 | 0.63 |
| A0A0N4SV40 | Plasminogen activator inhibitor 1 RNA-binding protein (Fragment) | 0.80 | 1.25 |
| Q62377 | U2 small nuclear ribonucleoprotein auxiliary factor 35 kDa subunit-related protein 2 | 0.84 | 1.27 |
| J3QPC8 | Troponin T, slow skeletal muscle | 1.04 | 1.30 |
| G3X8Q5 | Ceruloplasmin | 1.11 | 1.02 |
| O09061 | Proteasome subunit beta type-1 | 1.14 | 1.27 |
| Q5D098 | Proteasome subunit beta (EC 3.4.25.1) (Fragment) | 1.15 | 1.33 |
| Q9DCD8 | Proteasome subunit alpha type | 1.29 | 1.28 |
| A0A0R4J138 | Arylsulfatase B | 1.32 | 1.41 |
| P49722 | Proteasome subunit alpha type-2 | 1.52 | 1.34 |
| Q9EQ80 | NIF3-like protein 1 | 2.48 | 1.90 |
| Q548K2 | Alcohol dehydrogenase class 4 | 2.64 | 1.51 |
