# Supplementary material for: Altered histone abundance as a mode of ovotoxicity during 7,12-dimethylbenz[a]anthracene exposure with additive influence of obesity
Source: Biol Reprod. 2023 Oct 19;110(2):419–29. doi: 10.1093/biolre/ioad140 (PMC10873273; doi:10.1093/biolre/ioad140)
Supplement: supplemental_table_4_ioad140 [file supplemental_table_4_ioad140.docx]

**Supplemental Table 4.** **Impact of DMBA exposure on ovarian protein abundance in obese mice.** After 7 d of exposure to vehicle control or DMBA in obese mice, total ovarian proteome changes were quantified via LC-MS/MS. Proteins (120) altered (*P* < 0.05; n = 5) by DMBA exposure in obese mice are listed.

| **Uniprot ID** | **Protein names** | **Log2 (FC)** | | | **q-value** | | |
| --- | --- | --- | --- | --- | --- | --- | --- |
| Q920E5 | Farnesyl pyrophosphate synthase | | -2.39 | | 0.56 | | |
| Q3UB67 | Uncharacterized protein | | -2.00 | | 0.65 | | |
| P61205 | ADP-ribosylation factor 3 | | -1.71 | | 0.96 | | |
| A0A0G2JGD2 | Protein S100-A4 (Fragment) | | -1.69 | | 0.59 | | |
| P34914 | Bifunctional epoxide hydrolase 2 | | -1.64 | | 0.64 | | |
| P60122 | RuvB-like 1 | | -1.62 | | 0.62 | | |
| Q3UZI0 | Uncharacterized protein (Fragment) | | -1.57 | | 0.52 | | |
| Q8C2E1 | Uncharacterized protein | | -1.51 | | 0.64 | | |
| Q80YX1 | Tenascin | | -1.32 | | 0.56 | | |
| O88844 | Isocitrate dehydrogenase | | -1.32 | | 0.57 | | |
| Q8VCM7 | Fibrinogen gamma chain | | -1.32 | | 0.67 | | |
| P24815 | 3 beta-hydroxysteroid dehydrogenase | | -1.31 | | 0.52 | | |
| P52480 | Pyruvate kinase PKM | | -1.24 | | 0.58 | | |
| Q9D154 | Leukocyte elastase inhibitor A | | -1.22 | | 0.53 | | |
| A8DUK4 | Beta-globin | | -1.21 | | 0.52 | | |
| O55142 | 60S ribosomal protein L35a | | -1.20 | | 0.53 | | |
| P47738 | Aldehyde dehydrogenase | | -1.20 | | 0.52 | | |
| Q8VDC3 | Citrate hydro-lyase | | -1.19 | | 0.58 | | |
| Q04447 | Creatine kinase B-type | | -1.18 | | 0.63 | | |
| P13634 | Carbonic anhydrase 1 | | -1.15 | | 0.58 | | |
| A1BN54 | Alpha-actinin cytoskeletal isoform | | -1.08 | | 0.59 | | |
| Q9Z1Q5 | Chloride intracellular channel protein 1 | | -1.05 | | 0.51 | | |
| Q3UM20 | Uncharacterized protein | | -1.02 | | 0.58 | | |
| Q9QUI0 | Transforming protein RhoA | | -1.02 | | 0.60 | | |
| P19157 | Glutathione S-transferase P 1 | | -1.02 | | 0.58 | | |
| P05213 | Tubulin alpha-1B chain | | -0.96 | | 0.59 | | |
| P14069 | Protein S100-A6 | | -0.95 | | 0.63 | | |
| P30416 | Peptidyl-prolyl cis-trans isomerase | | -0.94 | | 0.57 | | |
| Q3UIJ3 | Uncharacterized protein | | -0.91 | | 0.59 | | |
| A0A1B0GSG5 | Ribonuclease inhibitor | | -0.86 | | 1.76 | | |
| P28474 | Alcohol dehydrogenase class-3 | | -0.84 | | 0.53 | | |
| Q64727 | Vinculin (Metavinculin) | | -0.84 | | 0.66 | | |
| P21981 | Protein-glutamine gamma-glutamyltransferase 2 | | | -0.83 | | 0.54 | |
| Q91YR9 | Prostaglandin reductase 1 (PRG-1) | | -0.81 | | 0.61 | | |
| Q3TIH8 | Uncharacterized protein | | -0.80 | | 0.52 | | |
| Q9CPV4 | Glyoxalase domain-containing protein 4 | | -0.74 | | 0.64 | | |
| O55029 | Coatomer subunit beta | | -0.68 | | 0.53 | | |
| P14824 | Annexin A6 (67 kDa calelectrin) | | -0.62 | | 0.58 | | |
| P99024 | Tubulin beta-5 chain | | -0.50 | | 0.51 | | |
| Q922R8 | Protein disulfide-isomerase A6 | | -0.47 | | 0.55 | | |
| P47754 | F-actin-capping protein subunit alpha-2 | | -0.46 | | 0.59 | | |
| Q8BQ02 | Uncharacterized protein | | -0.39 | | 0.59 | | |
| P09055 | Integrin beta-1 | | -0.36 | | 0.64 | | |
| P50543 | Protein S100-A11 (Calgizzarin) | | -0.30 | | 0.52 | | |
| Q91VW3 | SH3 domain-binding glutamic acid-rich-like protein 3 | | | 0.16 | | 0.58 | |
| Q9EQU5 | Protein SET (Phosphatase 2A inhibitor I2PP2A) | | 0.19 | | 0.64 | | |
| A0A668KLV9 | A-kinase anchor protein 12 | | 0.20 | | 0.52 | | |
| P83917 | Chromobox protein homolog 1 | | 0.22 | | 0.53 | | |
| Q99KJ8 | Dynactin subunit 2 | | 0.23 | | 0.53 | | |
| Q9DB15 | 39S ribosomal protein L12 | | 0.23 | | 0.51 | | |
| Q60668 | Heterogeneous nuclear ribonucleoprotein D0 | | 0.27 | | 0.68 | | |
| Q9JJU8 | SH3 domain-binding glutamic acid-rich-like protein | | 0.27 | | 0.59 | | |
| P10605 | Cathepsin B | | 0.29 | | 0.54 | | |
| Q3TWW8 | Serine/arginine-rich splicing factor 6 | | 0.29 | | 0.60 | | |
| P62774 | Myotrophin | | 0.30 | | 0.67 | | |
| Q9CR86 | Calcium-regulated heat stable protein 1 | | 0.30 | | 0.62 | | |
| Q63918 | Caveolae-associated protein 2 | | 0.30 | | 0.66 | | |
| Q64525 | Histone H2B type 2-B | | 0.31 | | 0.60 | | |
| B1AU75 | Nuclear autoantigenic sperm protein | | 0.31 | | 0.69 | | |
| Q8VCQ8 | Caldesmon 1 | | 0.32 | | 0.52 | | |
| P98078 | Disabled homolog 2 | | 0.33 | | 0.53 | | |
| Q8VE97 | Serine/arginine-rich splicing factor 4 | | 0.33 | | 0.54 | | |
| P10639 | Thioredoxin (Trx) | | 0.35 | | 0.52 | | |
| Q9JHL1 | Na(+)/H(+) exchange regulatory cofactor NHE-RF2 | | 0.36 | | 0.58 | | |
| G3UWX9 | Small ubiquitin-related modifier 3 | | 0.36 | | 0.58 | | |
| Q9JKB3 | Y-box-binding protein 3 | | 0.38 | | 0.62 | | |
| Q60932 | Voltage-dependent anion-selective channel protein 1 | | 0.39 | | 0.53 | | |
| Q571F9 | MKIAA4115 protein (Fragment) | | 0.39 | | 0.76 | | |
| Q9DCB8 | Iron-sulfur cluster assembly 2 homolog | | 0.41 | | 0.74 | | |
| P22599 | Alpha-1-antitrypsin 1-2 (AAT) | | 0.42 | | 0.56 | | |
| Q62005 | Zona pellucida sperm-binding protein 1 | | 0.42 | | 0.65 | | |
| A0A0N4SVS6 | Cellular nucleic acid-binding protein | | 0.42 | | 0.73 | | |
| Q3UX44 | Zona pellucida sperm-binding protein 2 | | 0.43 | | 0.70 | | |
| Q99LT0 | Protein dpy-30 homolog | | 0.43 | | 0.81 | | |
| Q9JMG7 | Hepatoma-derived growth factor-related protein 3 | | | 0.44 | | 0.57 | |
| Q8BGS2 | BolA-like protein 2 | | 0.45 | | 0.55 | | |
| P62077 | Mitochondrial import inner membrane translocase subunit Tim8 B | | | 0.45 | | 0.69 | |
| P32261 | Antithrombin-III (ATIII) | | 0.45 | | 0.52 | | |
| K3W4Q8 | Basigin | | 0.47 | | 0.60 | | |
| Q8BFS6 | Serine/threonine-protein phosphatase CPPED1 | | | 0.48 | | | 0.53 |
| Q9CQK7 | RWD domain-containing protein 1 | | | 0.49 | | | 0.59 |
| Q9JIX0 | Transcription and mRNA export factor ENY2 | | | 0.50 | | | 0.53 |
| Q8CHP5 | Partner of Y14 and mago | | | 0.51 | | | 0.60 |
| Q9JMG1 | Endothelial differentiation-related factor 1 | | | 0.51 | | | 0.58 |
| Q9EQC8 | Papillary Renal Cell carcinoma | | | 0.51 | | | 0.59 |
| E9PZM7 | SR-related CTD-associated factor 11 | | | 0.52 | | | 0.57 |
| D3Z134 | Protein MGARP | | | 0.52 | | | 0.56 |
| B2RUF0 | Y box protein 2 | | | 0.53 | | | 0.58 |
| Q69ZC8 | GPALPP motifs-containing protein 1 | | | 0.54 | | | 0.56 |
| P62311 | U6 snRNA-associated Sm-like protein LSm3 | | | 0.54 | | | 0.66 |
| E9QP00 | Transformer-2 protein homolog alpha | | | 0.54 | | | 0.51 |
| O09164 | Extracellular superoxide dismutase | | | 0.54 | | | 0.58 |
| Q9D6J6 | NADH dehydrogenase | | | 0.55 | | | 0.62 |
| P56375 | Acylphosphatase-2 | | | 0.55 | | | 0.59 |
| D3Z5B1 | Zinc finger, BED type-containing 5 (Fragment) | | | 0.58 | | | 0.70 |
| Q91ZA3 | Propionyl-CoA carboxylase alpha chain | | | 0.60 | | | 0.52 |
| P07758 | Alpha-1-antitrypsin 1-1 | | | 0.62 | | | 0.62 |
| Q3UDH7 | RRM domain-containing protein | | | 0.63 | | | 0.69 |
| Q80WJ7 | Protein LYRIC | | | 0.65 | | | 0.65 |
| F2Z455 | Four and a half LIM domains protein 3 | | | 0.66 | | | 0.66 |
| Q80DW0 | A14L protein | | | 0.67 | | | 0.53 |
| Q8CH18 | Cell division cycle and apoptosis regulator protein 1 | | | 0.67 | | | 0.52 |
| E9Q9E1 | Eukaryotic translation initiation factor 4 gamma 1 | | | 0.70 | | | 0.51 |
| P28574 | Protein max (Myc-associated factor X) | | | 0.75 | | | 0.59 |
| Q8VCG1 | Deoxyuridine 5'-triphosphate nucleotidohydrolase | | 0.77 | | 0.52 | | |
| Q60772 | Cyclin-dependent kinase 4 inhibitor C | | 0.80 | | 0.65 | | |
| Q61191 | Host cell factor 1 | | | 0.80 | | 0.54 | |
| A0A286YDB7 | Signal sequence receptor subunit alpha | | | 0.81 | | 0.53 | |
| Q8K2F8 | Protein LSM14 homolog A (Protein FAM61A) (RNA-associated protein 55A) (mRAP55A) | | | 0.84 | | 0.58 | |
| Q7TQH0 | Ataxin-2-like protein | | | 0.89 | | 0.72 | |
| Q8C5P7 | Testis development-related protein | | | 0.98 | | 0.52 | |
| P11404 | Fatty acid-binding protein, heart | | | 0.98 | | 0.57 | |
| Q8VI94 | 2'-5'-oligoadenylate synthase-like protein 1 | | | 0.98 | | 0.64 | |
| Q9Z0F7 | Gamma-synuclein (Persyn) | | | 1.05 | | 0.69 | |
| Q3UGS4 | Mapk-regulated corepressor-interacting protein 1 | | | 1.15 | | 0.90 | |
| Q6PDS4 | Frataxin intermediate form | | | 1.22 | | 0.65 | |
| Q07797 | Galectin-3-binding protein | | | 1.29 | | 0.68 | |
| Q9CPW2 | Ferredoxin-2, mitochondrial | | | 1.32 | | 0.66 | |
| Q9JHU2 | Palmdelphin | | | 1.37 | | 0.73 | |
| P61022 | Calcineurin B homologous protein 1 | | | 1.58 | | 0.58 | |
