# Supplementary material for: Altered histone abundance as a mode of ovotoxicity during 7,12-dimethylbenz[a]anthracene exposure with additive influence of obesity
Source: Biol Reprod. 2023 Oct 19;110(2):419–29. doi: 10.1093/biolre/ioad140 (PMC10873273; doi:10.1093/biolre/ioad140)
Supplement: supplemental_table_5_ioad140 [file supplemental_table_5_ioad140.docx]

**Supplemental Table 5.** Reproductive and DNA repair pathways affected (*P* < 0.05; n = 5) by DMBA exposure in obese mice.

| **Pathway** | **# of protein hits** | **Protein Names** | **Uniprot ID** |
| --- | --- | --- | --- |
| Gonadotropin-releasing hormone receptor pathway (P06664) | 4 | Tubulin alpha-1B chain | P05213 |
|  |  | Vinculin | Q64727 |
|  |  | Y-box-binding protein 3 | Q9JKB3 |
|  |  | Integrin beta-1 | P09055 |
| p53 pathway (P00059) | 2 | Small ubiquitin-related modifier 1 | P63166 |
|  |  | Cyclin-dependent kinase 4 inhibitor C | Q60772 |
