# Supplementary material for: Altered histone abundance as a mode of ovotoxicity during 7,12-dimethylbenz[a]anthracene exposure with additive influence of obesity
Source: Biol Reprod. 2023 Oct 19;110(2):419–29. doi: 10.1093/biolre/ioad140 (PMC10873273; doi:10.1093/biolre/ioad140)
Supplement: supplemental_table_6_ioad140 [file supplemental_table_6_ioad140.docx]

**Supplemental Table 6.**  **Impact of DMBA exposure on ovarian protein abundance in lean compared to obese mice.** After 7 d of exposure to vehicle control or DMBA in lean and obese mice, total ovarian proteome changes were quantified via LC-MS/MS. Proteins (634) altered (*P* < 0.05; n = 5) by DMBA in obese compared to lean mice are listed.

| **Uniprot ID** | **Protein names** | **Log2(FC)** | | **q-value** |
| --- | --- | --- | --- | --- |
| Q3UGR6 | Platelet-activating factor acetylhydrolase IB subunit alpha | | -5.77 | < 0.05 |
| P49722 | Proteasome subunit alpha type-2 | | -5.38 | < 0.05 |
| Q6NSR8 | Probable aminopeptidase NPEPL1 | | -5.19 | < 0.05 |
| Q9R1T2 | SUMO-activating enzyme subunit 1 (Ubiquitin-like 1-activating enzyme E1A) | | -4.95 | < 0.05 |
| Q8CAJ7 | Aspartyl aminopeptidase | | -4.94 | 0.074 |
| Q9QUM9 | Proteasome subunit alpha type-6 | | -4.89 | < 0.05 |
| P00493 | Hypoxanthine-guanine phosphoribosyltransferase | | -4.86 | < 0.05 |
| Q9CQ65 | S-methyl-5'-thioadenosine phosphorylase | | -4.85 | < 0.05 |
| P11352 | Glutathione peroxidase 1 | | -4.84 | < 0.05 |
| P17563 | Methanethiol oxidase (MTO) | | -4.80 | < 0.05 |
| P99026 | Proteasome subunit beta type-4 | | -4.80 | < 0.05 |
| Q9DCD8 | Proteasome subunit alpha type | | -4.70 | < 0.05 |
| P05201 | Aspartate aminotransferase, cytoplasmic (cAspAT) | | -4.64 | < 0.05 |
| Q3TN31 | Proteasome subunit alpha type | | -4.64 | < 0.05 |
| Q9DBB8 | Trans-1,2-dihydrobenzene-1,2-diol dehydrogenase | | -4.61 | 0.063 |
| Q9D2G2 | Dihydrolipoyllysine-residue succinyltransferase component of 2-oxoglutarate dehydrogenase complex | | -4.59 | < 0.05 |
| Q3TM67 | PUA domain-containing protein | | -4.52 | < 0.05 |
| P40142 | Transketolase (TK) (EC 2.2.1.1) | | -4.33 | < 0.05 |
| O08749 | Dihydrolipoyl dehydrogenase | | -4.32 | < 0.05 |
| P42125 | Enoyl-CoA delta isomerase 1 | | -4.32 | < 0.05 |
| Q9DBJ1 | Phosphoglycerate mutase 1 | | -4.14 | < 0.05 |
| Q9D819 | Inorganic pyrophosphatase | | -4.13 | < 0.05 |
| P62806 | Histone H4 | | -4.03 | < 0.05 |
| Q71V27 | Smooth muscle LIM protein | | -4.03 | < 0.05 |
| B1AU71 | DBF4-type zinc finger-containing protein 2 homolog (Fragment) | | -3.95 | < 0.05 |
| Q8JZM0 | Dimethyladenosine transferase 1, mitochondrial | | -3.94 | < 0.05 |
| Q9D172 | Glutamine amidotransferase-like class 1 domain-containing protein 3A | | -3.92 | < 0.05 |
| P56480 | ATP synthase subunit beta, mitochondrial | | -3.84 | < 0.05 |
| G3X8Q5 | Ceruloplasmin | | -3.84 | < 0.05 |
| Q8BWT1 | 3-ketoacyl-CoA thiolase | | -3.83 | < 0.05 |
| Q01853 | Transitional endoplasmic reticulum ATPase | | -3.81 | < 0.05 |
| Q9JHU9 | Inositol-3-phosphate synthase 1 | | -3.76 | < 0.05 |
| Q9R1P0 | Proteasome subunit alpha type-4 | | -3.74 | < 0.05 |
| F6QYF8 | Aminopeptidase (EC 3.4.11.-) (Fragment) | | -3.72 | < 0.05 |
| P84244 | Histone H3.3 | | -3.71 | < 0.05 |
| O55234 | Proteasome subunit beta type-5 | | -3.64 | < 0.05 |
| Q6PB51 | Coiled-coil domain-containing protein 117 | | -3.63 | < 0.05 |
| Q8K2B3 | Succinate dehydrogenase [ubiquinone] flavoprotein subunit | | -3.63 | < 0.05 |
| U5LP42 | Anti-H5N1 hemagglutinin monoclonal anitbody H5M9 heavy chain (Fragment) | | -3.61 | < 0.05 |
| Q8BIJ6 | Isoleucine--tRNA ligase | | -3.60 | 0.096 |
| P20108 | Thioredoxin-dependent peroxide reductase | | -3.56 | < 0.05 |
| O88958 | Glucosamine-6-phosphate isomerase 1 | | -3.48 | < 0.05 |
| P00329 | Alcohol dehydrogenase 1 | | -3.47 | < 0.05 |
| Q9DCC4 | Pyrroline-5-carboxylate reductase 3 | | -3.47 | < 0.05 |
| Q99L27 | GMP reductase 2 (GMPR 2) | | -3.46 | < 0.05 |
| A0A0R4J107 | Acyl-peptide hydrolase | | -3.43 | 0.053 |
| Q9CPY7 | Cytosol aminopeptidase | | -3.42 | < 0.05 |
| O09061 | Proteasome subunit beta type-1 | | -3.36 | < 0.05 |
| B2RY90 | Isoc2a protein | | -3.32 | < 0.05 |
| A0A0R4J131 | Biotinidase | | -3.30 | < 0.05 |
| P06745 | Glucose-6-phosphate isomerase (GPI) | | -3.21 | < 0.05 |
| A0A0R4J138 | Arylsulfatase B | | -3.20 | < 0.05 |
| D3U0D7 | Kidney specific expressed protein 1 | | -3.18 | < 0.05 |
| P42208 | Septin-2 (Neural precursor cell expressed developmentally down-regulated protein 5) | | -3.16 | < 0.05 |
| Q9QZQ8 | Core histone macro-H2A.1 (Histone macroH2A1) | | -3.14 | < 0.05 |
| P59242 | Cingulin | | -3.13 | < 0.05 |
| Q5D098 | Proteasome subunit beta (EC 3.4.25.1) (Fragment) | | -3.13 | < 0.05 |
| P62315 | Small nuclear ribonucleoprotein Sm D1 (Sm-D1) | | -3.08 | < 0.05 |
| Q9Z2U1 | Proteasome subunit alpha type-5 (EC 3.4.25.1) | | -3.06 | < 0.05 |
| Q03265 | ATP synthase subunit alpha, mitochondrial (ATP synthase F1 subunit alpha) | | -3.04 | < 0.05 |
| Q9DCT2 | NADH dehydrogenase [ubiquinone] iron-sulfur protein 3 | | -3.02 | < 0.05 |
| Q9R1P3 | Proteasome subunit beta type-2 (EC 3.4.25.1) | | -2.99 | < 0.05 |
| Q78PY7 | Staphylococcal nuclease domain-containing protein 1 | | -2.99 | < 0.05 |
| P17918 | Proliferating cell nuclear antigen (PCNA) | | -2.89 | < 0.05 |
| E9PZF0 | Nucleoside diphosphate kinase | | -2.88 | < 0.05 |
| Q543K9 | Purine nucleoside phosphorylase | | -2.87 | 0.079 |
| Q8C622 | Histone domain-containing protein | | -2.83 | < 0.05 |
| P24549 | Retinal dehydrogenase 1 (RALDH 1) | | -2.82 | < 0.05 |
| Q920E5 | Farnesyl pyrophosphate synthase (FPP synthase) | | -2.78 | 0.074 |
| Q9Z1Z2 | Serine-threonine kinase receptor-associated protein (UNR-interacting protein) | | -2.77 | < 0.05 |
| Q52KG9 | Chaperonin containing Tcp1, subunit 6a (Zeta) | | -2.76 | < 0.05 |
| Q8VDC3 | Citrate hydro-lyase | | -2.73 | 0.071 |
| D3YTP8 | U6 snRNA-associated Sm-like protein LSm4 (Fragment) | | -2.73 | < 0.05 |
| A2AFQ2 | 3-hydroxyacyl-CoA dehydrogenase type-2 | | -2.72 | < 0.05 |
| P28474 | Alcohol dehydrogenase class-3 | | -2.71 | < 0.05 |
| P62317 | Small nuclear ribonucleoprotein Sm D2 (Sm-D2) | | -2.70 | < 0.05 |
| Q9JII5 | DAZ-associated protein 1 | | -2.69 | < 0.05 |
| P32067 | Lupus La protein homolog | | -2.66 | < 0.05 |
| Q9WUL7 | ADP-ribosylation factor-like protein 3 | | -2.64 | < 0.05 |
| Q921I1 | Serotransferrin (Transferrin) | | -2.63 | < 0.05 |
| Q921S3 | Malic enzyme | | -2.61 | 0.054 |
| A0A1B0GSX0 | L-lactate dehydrogenase | | -2.61 | < 0.05 |
| P62307 | Small nuclear ribonucleoprotein F | | -2.60 | < 0.05 |
| P16125 | L-lactate dehydrogenase B chain | | -2.59 | < 0.05 |
| P56399 | Ubiquitin carboxyl-terminal hydrolase 5 | | -2.57 | < 0.05 |
| Q3U3C2 | Epididymal secretory protein E1 | | -2.56 | < 0.05 |
| Q61553 | Fascin (Singed-like protein) | | -2.53 | < 0.05 |
| Q3UBJ6 | Eukaryotic translation initiation factor 6 | | -2.51 | < 0.05 |
| P46412 | Glutathione peroxidase 3 | | -2.51 | < 0.05 |
| P05202 | Aspartate aminotransferase | | -2.49 | < 0.05 |
| A0A0R4J083 | Long-chain specific acyl-CoA dehydrogenase | | -2.49 | < 0.05 |
| P97315 | Cysteine and glycine-rich protein 1 | | -2.48 | < 0.05 |
| P01027 | Complement C3 (HSE-MSF) | | -2.46 | < 0.05 |
| Q9R1P4 | Proteasome subunit alpha type-1 | | -2.45 | < 0.05 |
| Q3UXL1 | Aldo-keto reductase family 1 | | -2.44 | < 0.05 |
| Q8K023 | Aldo-keto reductase family 1 member C18 | | -2.43 | < 0.05 |
| Q9CZ42 | ATP-dependent (S)-NAD(P)H-hydrate dehydratase | | -2.43 | < 0.05 |
| P14069 | Protein S100-A6 (5B10) (Calcyclin) | | -2.40 | < 0.05 |
| Q3U8H8 | NTF2 domain-containing protein | | -2.32 | < 0.05 |
| Q3U8W0 | Protein-serine/threonine phosphatase | | -2.32 | < 0.05 |
| Q80YX1 | Tenascin (TN) (Hexabrachion) | | -2.28 | < 0.05 |
| Q8VHC3 | Selenoprotein M (SelM) | | -2.28 | < 0.05 |
| Q921M3 | Splicing factor 3B subunit 3 | | -2.23 | < 0.05 |
| P63330 | Serine/threonine-protein phosphatase 2A catalytic subunit alpha isoform | | -2.19 | 0.071 |
| P61205 | ADP-ribosylation factor 3 | | -2.17 | < 0.05 |
| Q61171 | Peroxiredoxin-2 | | -2.16 | < 0.05 |
| C5H0E8 | Caspase-6 (CASP-6) | | -2.12 | < 0.05 |
| P21981 | Protein-glutamine gamma-glutamyltransferase 2 | | -2.11 | < 0.05 |
| Q8C153 | Tr-type G domain-containing protein | | -2.08 | < 0.05 |
| P14152 | Malate dehydrogenase, cytoplasmic | | -2.06 | < 0.05 |
| Q9CPT4 | Myeloid-derived growth factor (MYDGF) | | -2.04 | < 0.05 |
| F8WIV2 | Serine (or cysteine) peptidase inhibitor | | -2.04 | < 0.05 |
| Q3TKC5 | Amidophosphoribosyltransferase | | -2.02 | < 0.05 |
| Q6GT24 | Peroxiredoxin-6 | | -1.99 | 0.053 |
| Q8VEE0 | Ribulose-phosphate 3-epimerase | | -1.99 | < 0.05 |
| O70433 | Four and a half LIM domains protein 2 | | -1.99 | < 0.05 |
| A0A0G2JGD2 | Protein S100-A4 (Fragment) | | -1.98 | < 0.05 |
| Q9CQF7 | Prefoldin 1 (Prefoldin subunit 1) | | -1.98 | < 0.05 |
| P62889 | 60S ribosomal protein L30 | | -1.98 | < 0.05 |
| Q9WVQ5 | Methylthioribulose-1-phosphate dehydratase | | -1.95 | < 0.05 |
| J3QPC8 | Troponin T | | -1.87 | < 0.05 |
| D3Z5G7 | Carboxylic ester hydrolase | | -1.86 | < 0.05 |
| Q9DCZ1 | GMP reductase 1 | | -1.86 | < 0.05 |
| Q9R1P1 | Proteasome subunit beta type-3 | | -1.85 | < 0.05 |
| Q642K0 | Myl6 protein | | -1.83 | < 0.05 |
| A0A0N4SV40 | Plasminogen activator inhibitor 1 RNA-binding protein (Fragment) | | -1.81 | < 0.05 |
| E9Q2S9 | Coiled-coil domain-containing protein 43 | | -1.80 | < 0.05 |
| P08249 | Malate dehydrogenase | | -1.80 | < 0.05 |
| C5H0E8 | Rap1A-retro1 | | -1.79 | < 0.05 |
| P63101 | 14-3-3 protein zeta/delta | | -1.78 | < 0.05 |
| P62259 | 14-3-3 protein epsilon | | -1.78 | < 0.05 |
| Q9DCN2 | NADH-cytochrome b5 reductase 3 (B5R) | | -1.78 | < 0.05 |
| P10493 | Nidogen-1 (NID-1) (Entactin) | | -1.76 | < 0.05 |
| P35700 | Peroxiredoxin-1 (EC 1.11.1.24) | | -1.75 | < 0.05 |
| P05064 | Fructose-bisphosphate aldolase A | | -1.71 | 0.055 |
| O08807 | Peroxiredoxin-4 | | -1.71 | < 0.05 |
| A0A1B0GSG5 | Ribonuclease inhibitor (Ribonuclease/angiogenin inhibitor 1) | | -1.70 | < 0.05 |
| P26043 | Radixin (ESP10) | | -1.67 | < 0.05 |
| Q9CQ60 | 6-phosphogluconolactonase | | -1.66 | 0.093 |
| P26041 | Moesin (Membrane-organizing extension spike protein) | | -1.65 | < 0.05 |
| Q3TRH8 | F-actin-capping protein subunit beta | | -1.64 | < 0.05 |
| Q60692 | Proteasome subunit beta type-6 | | -1.64 | 0.077 |
| Q3U1J4 | DNA damage-binding protein 1 | | -1.59 | < 0.05 |
| Q7M6Z4 | Kinesin-like protein KIF27 | | -1.57 | < 0.05 |
| Q62348 | Translin | | -1.56 | 0.059 |
| Q3UYH9 | Alpha-1,4 glucan phosphorylase | | -1.54 | < 0.05 |
| O35855 | Branched-chain-amino-acid aminotransferase | | -1.54 | < 0.05 |
| O09131 | Glutathione S-transferase omega-1 | | -1.54 | < 0.05 |
| P68254 | 14-3-3 protein theta (14-3-3 protein tau) | | -1.53 | < 0.05 |
| Q6ZVL3 | cDNA FLJ42424 fis, clone BLADE2004089 | | -1.52 | 0.055 |
| Q9WVJ3 | Carboxypeptidase Q | | -1.50 | < 0.05 |
| P09671 | Superoxide dismutase [Mn] | | -1.50 | < 0.05 |
| Q6TDG6 | Hypoxanthine guanine phosphoribosyl transferase (Fragment) | | -1.48 | < 0.05 |
| P63168 | Dynein light chain 1, cytoplasmic | | -1.46 | < 0.05 |
| P97807 | Fumarate hydratase, mitochondrial | | -1.45 | < 0.05 |
| A0A571BEC9 | Perilipin-4 | | -1.44 | < 0.05 |
| A0A0R4J2B2 | BTB/POZ domain-containing protein KCTD12 | | -1.44 | < 0.05 |
| E0CXJ3 | Eukaryotic translation initiation factor 2 subunit 2 (Fragment) | | -1.44 | < 0.05 |
| Q8VDM6 | Heterogeneous nuclear ribonucleoprotein U-like protein 1 | | -1.44 | < 0.05 |
| Q9WV54 | Acid ceramidase | | -1.44 | < 0.05 |
| Q8CFZ6 | C-type lectin domain family 3 | | -1.42 | < 0.05 |
| P61982 | 14-3-3 protein gamma | | -1.42 | < 0.05 |
| P23953 | Carboxylesterase 1C | | -1.42 | < 0.05 |
| E9Q5L2 | Inter alpha-trypsin inhibitor | | -1.38 | < 0.05 |
| P58044 | Isopentenyl-diphosphate Delta-isomerase 1 | | -1.38 | < 0.05 |
| P34914 | Bifunctional epoxide hydrolase 2 | | -1.38 | < 0.05 |
| Q04447 | Creatine kinase B-type | | -1.37 | < 0.05 |
| Q61838 | Pregnancy zone protein | | -1.37 | < 0.05 |
| Q9CQ62 | 2,4-dienoyl-CoA reductase | | -1.37 | 0.063 |
| Q3TI61 | 26S proteasome non-ATPase regulatory subunit 2 | | -1.36 | < 0.05 |
| P68510 | 14-3-3 protein eta | | -1.35 | 0.093 |
| P21614 | Vitamin D-binding protein | | -1.33 | < 0.05 |
| P62814 | V-type proton ATPase subunit B | | -1.33 | < 0.05 |
| Q3TBM1 | Alpha-mannosidase | | -1.33 | 0.056 |
| P07901 | Heat shock protein HSP 90-alpha | | -1.33 | < 0.05 |
| P50247 | Adenosylhomocysteinase (AdoHcyase) | | -1.33 | < 0.05 |
| Q9DCW4 | Electron transfer flavoprotein subunit beta (Beta-ETF) | | -1.32 | 0.097 |
| A0A0R4J0Z1 | Protein disulfide-isomerase A4 | | -1.29 | < 0.05 |
| Q9Z0K8 | Pantetheinase | | -1.25 | 0.076 |
| Q9CPU0 | Lactoylglutathione lyase | | -1.22 | < 0.05 |
| Q93092 | Transaldolase | | -1.20 | < 0.05 |
| O70318 | Band 4.1-like protein 2 | | -1.20 | < 0.05 |
| Q61233 | Plastin-2 (65 kDa macrophage protein) | | -1.20 | < 0.05 |
| P63038 | 60 kDa heat shock protein | | -1.19 | < 0.05 |
| D3Z4U0 | Zinc finger Ran-binding domain-containing protein 2 | | -1.18 | < 0.05 |
| Q7TMQ1 | Gap junction protein | | -1.16 | < 0.05 |
| Q9CQJ6 | Density-regulated protein (DRP) | | -1.13 | < 0.05 |
| Q08879 | Fibulin-1 (FIBL-1) | | -1.11 | < 0.05 |
| Q9DBG5 | Perilipin-3 | | -1.11 | 0.072 |
| Q9Z1N5 | Spliceosome RNA helicase Ddx39b | | -1.10 | 0.081 |
| P14824 | Annexin A6 (67 kDa calelectrin) | | -1.10 | < 0.05 |
| Q8BL66 | Early endosome antigen 1 | | -1.10 | 0.065 |
| Q9R0P5 | Destrin (Actin-depolymerizing factor) | | -1.09 | < 0.05 |
| D3Z5P0 | Serine/threonine-protein kinase BRSK1 | | -1.07 | < 0.05 |
| Q9D6J6 | NADH dehydrogenase [ubiquinone] | | -1.06 | < 0.05 |
| P30416 | Peptidyl-prolyl cis-trans isomerase FKBP4 | | -1.03 | < 0.05 |
| Q9CRB2 | H/ACA ribonucleoprotein complex subunit 2 | | -1.02 | < 0.05 |
| B1B0C7 | Basement membrane-specific heparan sulfate proteoglycan core protein | | -1.00 | < 0.05 |
| A0A498WGD8 | Thioredoxin-like protein 1 | | -0.98 | 0.068 |
| Q9QUI0 | Transforming protein RhoA | | -0.98 | < 0.05 |
| Q99L75 | Heat shock 70 kDa protein 4 | | -0.93 | < 0.05 |
| P11499 | Heat shock protein HSP 90-beta | | -0.92 | < 0.05 |
| Q3UAF6 | Uncharacterized protein | | -0.90 | 0.055 |
| Q9CQ19 | Myosin regulatory light polypeptide 9 | | -0.90 | < 0.05 |
| Q3UE99 | Uncharacterized protein (Fragment) | | -0.90 | < 0.05 |
| P47738 | Aldehyde dehydrogenase | | -0.90 | < 0.05 |
| P99029 | Peroxiredoxin-5, mitochondrial | | -0.89 | 0.081 |
| Q3U9Q8 | Actin-depolymerizing factor (Brevin) | | -0.87 | 0.093 |
| A0A0A0MQF6 | Glyceraldehyde-3-phosphate dehydrogenase | | -0.86 | 0.051 |
| Q00519 | Xanthine dehydrogenase/oxidase | | -0.86 | < 0.05 |
| P24369 | Peptidyl-prolyl cis-trans isomerase B | | -0.80 | 0.058 |
| Q9QY76 | Vesicle-associated membrane protein-associated protein B | | -0.79 | 0.065 |
| P09055 | Integrin beta-1 (Fibronectin receptor subunit beta) | | -0.73 | < 0.05 |
| B2M1R6 | Heterogeneous nuclear ribonucleoprotein K | | -0.69 | < 0.05 |
| Q9Z0P4 | Paralemmin-1 (Paralemmin) | | -0.69 | < 0.05 |
| P05213 | Tubulin alpha-1B chain (Alpha-tubulin 2) | | -0.68 | < 0.05 |
| A0A0G2JGW9 | Far upstream element-binding protein 1 | | -0.65 | < 0.05 |
| Q3U630 | Threonyl-tRNA synthetase | | -0.62 | 0.077 |
| P11031 | Activated RNA polymerase II transcriptional coactivator p15 | | -0.59 | 0.071 |
| Q3U6P5 | RRM domain-containing protein | | -0.55 | 0.089 |
| Q8CD23 | Nucleolin | | -0.55 | < 0.05 |
| H9KV15 | Protein SON | | -0.52 | 0.053 |
| Q8CGP5 | Histone H2A type 1-F | | -0.43 | 0.085 |
| P28667 | MARCKS-related protein (Brain protein F52) | | -0.38 | < 0.05 |
| P26645 | Myristoylated alanine-rich C-kinase substrate (MARCKS) | | -0.24 | < 0.05 |
| Q8R0F8 | Acylpyruvase FAHD1, mitochondrial (EC 3.7.1.5) | | -0.07 | < 0.05 |
| Q9JL35 | High mobility group nucleosome-binding domain-containing protein 5 | | 0.23 | < 0.05 |
| P08228 | Superoxide dismutase [Cu-Zn] | | 0.34 | 0.086 |
| Q8CCS6 | Polyadenylate-binding protein 2 (PABP-2) | | 0.34 | 0.071 |
| Q62418 | Drebrin-like protein (Actin-binding protein 1) | | 0.36 | < 0.05 |
| P09411 | Phosphoglycerate kinase 1 | | 0.37 | 0.078 |
| Q9EQU5 | Protein SET (Phosphatase 2A inhibitor I2PP2A) | | 0.37 | < 0.05 |
| Q6P8I4 | PEST proteolytic signal-containing nuclear protein (PCNP) | | 0.38 | 0.096 |
| Q9EQS3 | c-Myc-binding protein (Associate of Myc 1) | | 0.38 | 0.065 |
| Q4V9X9 | Rpl23a protein (Fragment) | | 0.38 | < 0.05 |
| Q6PAM1 | Alpha-taxilin | | 0.39 | 0.059 |
| Q3UHX2 | 28 kDa heat- and acid-stable phosphoprotein | | 0.39 | 0.085 |
| Q9D824 | Pre-mRNA 3'-end-processing factor FIP1 | | 0.40 | < 0.05 |
| Q9DAK9 | 14 kDa phosphohistidine phosphatase | | 0.41 | 0.086 |
| Q3TIU3 | Alpha-2-HS-glycoprotein | | 0.42 | 0.091 |
| Q3UEI6 | HABP4_PAI-RBP1 domain-containing protein | | 0.42 | < 0.05 |
| E9PXX7 | Thioredoxin domain-containing protein 5 | | 0.42 | 0.094 |
| Q3UZG3 | Uncharacterized protein | | 0.43 | 0.054 |
| P48678 | Prelamin-A/C [Cleaved into: Lamin-A/C] | | 0.43 | < 0.05 |
| P97822 | Acidic leucine-rich nuclear phosphoprotein 32 family member E | | 0.44 | < 0.05 |
| P97450 | ATP synthase-coupling factor 6 | | 0.45 | < 0.05 |
| P14211 | Calreticulin (CRP55) | | 0.45 | < 0.05 |
| P51859 | Hepatoma-derived growth factor (HDGF) | | 0.47 | < 0.05 |
| A0A087WQ25 | Kinectin | | 0.47 | 0.083 |
| Q9ERE7 | LRP chaperone MESD (LDLR chaperone MESD) | | 0.48 | 0.097 |
| P34022 | Ran-specific GTPase-activating protein | | 0.49 | < 0.05 |
| Q3TE95 | Uncharacterized protein | | 0.49 | < 0.05 |
| E9Q456 | Tropomyosin alpha-1 chain | | 0.50 | 0.054 |
| B1AU75 | Nuclear autoantigenic sperm protein | | 0.50 | < 0.05 |
| P97352 | Protein S100-A13 (S100 calcium-binding protein A13) | | 0.51 | 0.083 |
| Q4VAA2 | Protein CDV3 (Carnitine deficiency-associated protein 3) | | 0.51 | < 0.05 |
| P55302 | Alpha-2-macroglobulin receptor-associated protein | | 0.51 | < 0.05 |
| Q60668 | Heterogeneous nuclear ribonucleoprotein D0 | | 0.51 | < 0.05 |
| Q8WTY4 | Anamorsin (Cytokine-induced apoptosis inhibitor 1) | | 0.52 | 0.080 |
| Q99LX0 | Parkinson disease protein 7 homolog (Maillard deglycase) | | 0.52 | 0.092 |
| Q9JLQ0 | CD2-associated protein | | 0.53 | < 0.05 |
| P57776 | Elongation factor 1-delta | | 0.53 | 0.089 |
| P62852 | 40S ribosomal protein S25 | | 0.54 | 0.076 |
| A0A0R4J038 | Bradykinin | | 0.54 | 0.066 |
| Q3TSY9 | RRM domain-containing protein | | 0.54 | < 0.05 |
| F6WGR1 | Serine/threonine-protein kinase pim-2 (Fragment) | | 0.54 | < 0.05 |
| Q8BGC0 | HIV Tat-specific factor 1 homolog | | 0.54 | < 0.05 |
| Q8BTI8 | Serine/arginine repetitive matrix protein 2 | | 0.54 | 0.054 |
| Q64433 | 10 kDa heat shock protein | | 0.55 | 0.094 |
| Q3TE63 | Peptidyl-prolyl cis-trans isomerase | | 0.55 | < 0.05 |
| P31786 | Acyl-CoA-binding protein | | 0.56 | < 0.05 |
| D3Z134 | Protein MGARP | | 0.56 | < 0.05 |
| P27546 | Microtubule-associated protein 4 (MAP-4) | | 0.57 | < 0.05 |
| D3YXK2 | Scaffold attachment factor B1 (SAF-B1) | | 0.57 | < 0.05 |
| Q8CBE6 | Alpha-dystroglycan (Beta-dystroglycan) | | 0.57 | < 0.05 |
| P49817 | Caveolin-1 | | 0.57 | < 0.05 |
| G5E8R8 | UBX domain-containing protein 7 | | 0.57 | 0.083 |
| Q9CQ45 | Neudesin (Neuron-derived neurotrophic factor) | | 0.58 | < 0.05 |
| Q91XT3 | Stathmin | | 0.59 | < 0.05 |
| O70251 | Elongation factor 1-beta (EF-1-beta) | | 0.60 | < 0.05 |
| Q8CD09 | LIM zinc-binding domain-containing protein | | 0.60 | < 0.05 |
| A0A338P6F6 | High mobility group AT-hook protein 1 | | 0.60 | 0.090 |
| Q8BH97 | Reticulocalbin-3 | | 0.60 | < 0.05 |
| B9EKJ7 | Tankyrase 1 binding protein 1 | | 0.60 | < 0.05 |
| Q569Z6 | Thyroid hormone receptor-associated protein 3 | | 0.61 | < 0.05 |
| Q9DB15 | 39S ribosomal protein L12, mitochondrial | | 0.61 | < 0.05 |
| Q80ZU5 | Coiled-coil domain-containing protein 181 | | 0.61 | 0.055 |
| O35639 | Annexin A3 (35-alpha calcimedin) | | 0.61 | 0.051 |
| A0A0A6YW06 | Protein enabled homolog | | 0.61 | < 0.05 |
| Q9DBP5 | UMP-CMP kinase | | 0.61 | < 0.05 |
| Q8C1W9 | Uncharacterized protein | | 0.61 | < 0.05 |
| O70456 | 14-3-3 protein sigma (Stratifin) | | 0.62 | < 0.05 |
| P18760 | Cofilin-1 (Cofilin, non-muscle isoform) | | 0.62 | < 0.05 |
| P48024 | Eukaryotic translation initiation factor 1 (eIF1) | | 0.63 | 0.075 |
| O88569 | Heterogeneous nuclear ribonucleoproteins A2/B1 | | 0.63 | < 0.05 |
| Q3TJG6 | CS domain-containing protein | | 0.64 | < 0.05 |
| P68040 | Receptor of activated protein C kinase 1 (12-3) | | 0.64 | < 0.05 |
| Q3U781 | RRM domain-containing protein | | 0.64 | < 0.05 |
| A6H663 | BCL2-associated athanogene 3 | | 0.65 | 0.082 |
| A0A0R4J1N9 | Transcription factor A, mitochondrial | | 0.65 | 0.064 |
| A0A286YDA2 | Nucleolar and coiled-body phosphoprotein 1 | | 0.65 | < 0.05 |
| Q3U0V1 | Far upstream element-binding protein 2 | | 0.66 | < 0.05 |
| P32261 | Antithrombin-III (ATIII) (Serpin C1) | | 0.67 | 0.080 |
| Q925B0 | PRKC apoptosis WT1 regulator protein | | 0.67 | < 0.05 |
| P70296 | Phosphatidylethanolamine-binding protein 1 | | 0.67 | 0.057 |
| Q9R0P9 | Ubiquitin carboxyl-terminal hydrolase isozyme L1 | | 0.67 | 0.055 |
| Q9QZ23 | NFU1 iron-sulfur cluster scaffold homolog | | 0.67 | < 0.05 |
| Q61686 | Chromobox protein homolog 5 | | 0.67 | 0.052 |
| O88271 | Craniofacial development protein 1 | | 0.68 | < 0.05 |
| Q6W8Q3 | Purkinje cell protein 4-like protein 1 | | 0.68 | < 0.05 |
| Q9DCL8 | Protein phosphatase inhibitor 2 | | 0.68 | < 0.05 |
| Q80TU6 | MKIAA0670 protein (Fragment) | | 0.68 | < 0.05 |
| P20029 | Endoplasmic reticulum chaperone BiP protein family A member 5 | | 0.68 | < 0.05 |
| Q571F9 | MKIAA4115 protein (Fragment) | | 0.70 | < 0.05 |
| P99024 | Tubulin beta-5 chain | | 0.70 | < 0.05 |
| Q69ZX3 | MKIAA0866 protein (Fragment) | | 0.70 | 0.073 |
| Q3UVN5 | NSFL1 cofactor p47 (p97 cofactor p47) | | 0.70 | < 0.05 |
| Q3U422 | Complex I-9kD (NADH dehydrogenase | | 0.70 | < 0.05 |
| Q99J29 | Carboxypeptidase | | 0.70 | 0.092 |
| Q8R5G0 | Nidogen 2 protein | | 0.71 | < 0.05 |
| P60824 | Cold-inducible RNA-binding protein | | 0.71 | 0.077 |
| Q91WG2 | Rab GTPase-binding effector protein 2 | | 0.72 | < 0.05 |
| B2RRI4 | Adam17 protein | | 0.72 | 0.051 |
| P70445 | Eukaryotic translation initiation factor 4E-binding protein 2 | | 0.72 | 0.081 |
| Q9DBR7 | Protein phosphatase 1 regulatory subunit 12A | | 0.72 | < 0.05 |
| O88811 | Signal transducing adapter molecule 2 | | 0.72 | < 0.05 |
| Q7TNV0 | Protein DEK | | 0.72 | < 0.05 |
| Q6P1B9 | Bin1 protein (Myc box-dependent-interacting protein 1) | | 0.72 | < 0.05 |
| A2AA85 | SUZ domain-containing protein 1 (Fragment) | | 0.72 | 0.056 |
| P62897 | Cytochrome c, somatic | | 0.73 | < 0.05 |
| Q9DBB9 | Carboxypeptidase N subunit 2 | | 0.73 | < 0.05 |
| Q922J3 | CAP-Gly domain-containing linker protein 1 | | 0.73 | 0.059 |
| F6RDI8 | Serine and arginine-rich-splicing factor 11 (Fragment) | | 0.73 | < 0.05 |
| Q60605 | Myosin light polypeptide 6 | | 0.74 | < 0.05 |
| Q3UXU0 | Uncharacterized protein | | 0.74 | < 0.05 |
| Q8BSH9 | Nucleosome assembly protein 1-like 1 | | 0.74 | 0.078 |
| Q3UF30 | Calpactin I light chain | | 0.75 | < 0.05 |
| P63166 | Small ubiquitin-related modifier 1 (SUMO-1) | | 0.76 | 0.084 |
| Q05186 | Reticulocalbin-1 | | 0.77 | < 0.05 |
| Q9CR86 | Calcium-regulated heat stable protein 1 | | 0.77 | < 0.05 |
| Q9JHJ0 | Tropomodulin-3 (Ubiquitous tropomodulin) | | 0.77 | 0.062 |
| Q9CQK7 | RWD domain-containing protein 1 | | 0.77 | < 0.05 |
| Q62261 | Spectrin beta chain, non-erythrocytic 1 | | 0.78 | 0.071 |
| Q9D8S9 | BolA-like protein 1 | | 0.78 | < 0.05 |
| Q9D7P6 | Iron-sulfur cluster assembly enzyme ISCU | | 0.78 | 0.057 |
| Q6PGL7 | WASH complex subunit 2 | | 0.78 | 0.079 |
| Q61576 | Peptidyl-prolyl cis-trans isomerase FKBP10 | | 0.79 | 0.055 |
| A2AVR9 | Dynein light chain roadblock | | 0.79 | 0.088 |
| P55937 | Golgin subfamily A member 3 | | 0.79 | < 0.05 |
| Q5EBG8 | Uncharacterized protein C1orf50 homolog | | 0.80 | < 0.05 |
| H7BWX9 | Small ubiquitin-related modifier 2 | | 0.80 | < 0.05 |
| P10605 | Cathepsin B | | 0.81 | < 0.05 |
| Q80SZ7 | Guanine nucleotide-binding protein G | | 0.81 | 0.059 |
| O35326 | Serine/arginine-rich splicing factor 5 | | 0.81 | < 0.05 |
| O35381 | Acidic leucine-rich nuclear phosphoprotein 32 family member A | | 0.81 | < 0.05 |
| Q9CYA0 | Protein disulfide isomerase Creld2 | | 0.81 | < 0.05 |
| Q5SUH7 | Clathrin interactor 1 | | 0.82 | < 0.05 |
| Q8BMS1 | Trifunctional enzyme subunit alpha | | 0.83 | 0.076 |
| J3QJW3 | Calcium-dependent secretion activator 1 | | 0.83 | 0.071 |
| Q8BIQ5 | Cleavage stimulation factor subunit 2 | | 0.83 | < 0.05 |
| P46935 | E3 ubiquitin-protein ligase NEDD4 | | 0.84 | < 0.05 |
| Q61990 | Poly(rC)-binding protein 2 | | 0.85 | 0.085 |
| Q8R317 | Ubiquilin-1 | | 0.85 | 0.062 |
| Q62446 | Peptidyl-prolyl cis-trans isomerase FKBP3 | | 0.85 | < 0.05 |
| Q63918 | Caveolae-associated protein 2 | | 0.85 | < 0.05 |
| D3Z5B1 | Zinc finger, BED type-containing 5 (Fragment) | | 0.86 | < 0.05 |
| O35226 | 26S proteasome non-ATPase regulatory subunit 4 | | 0.86 | < 0.05 |
| Q9JKB3 | Y-box-binding protein 3 | | 0.87 | < 0.05 |
| P28184 | Metallothionein-3 (MT-3) | | 0.88 | < 0.05 |
| Q9D8B3 | Charged multivesicular body protein 4b | | 0.89 | 0.087 |
| Q6NZB0 | DnaJ homolog subfamily C member 8 | | 0.89 | < 0.05 |
| E9PV44 | ATP synthase F1 subunit epsilon | | 0.89 | 0.095 |
| P46664 | Adenylosuccinate synthetase isozyme 2 | | 0.89 | < 0.05 |
| A0A087WR57 | Death-associated protein 1 | | 0.90 | < 0.05 |
| Q8CGN5 | Perilipin-1 (Lipid droplet-associated protein) | | 0.91 | < 0.05 |
| Q9D8X2 | Coiled-coil domain-containing protein 124 | | 0.91 | 0.052 |
| G5E8V8 | Phosphorylated adapter RNA export protein | | 0.91 | < 0.05 |
| Q9JKR6 | Hypoxia up-regulated protein 1 | | 0.91 | < 0.05 |
| P16546 | Spectrin alpha chain, non-erythrocytic 1 | | 0.91 | < 0.05 |
| Q8C0W0 | Thymosin beta 15b-like | | 0.91 | < 0.05 |
| P27773 | Protein disulfide-isomerase A3 | | 0.91 | < 0.05 |
| P51885 | Lumican (Keratan sulfate proteoglycan lumican) | | 0.92 | < 0.05 |
| Q8CHP5 | Partner of Y14 and mago | | 0.92 | < 0.05 |
| Q8R409 | Protein HEXIM1 (Cardiac lineage protein 1) | | 0.93 | < 0.05 |
| P28481 | Collagen alpha-1(II) chain | | 0.93 | 0.055 |
| Q9Z1D1 | Eukaryotic translation initiation factor 3 subunit G | | 0.93 | < 0.05 |
| Q3TDX7 | Extracellular matrix protein 1 | | 0.93 | < 0.05 |
| Q6PGH2 | Jupiter microtubule associated homolog 2 | | 0.93 | < 0.05 |
| Q9CPR4 | 60S ribosomal protein L17 | | 0.94 | < 0.05 |
| Q3TFP8 | Cytochrome b5 heme-binding domain-containing protein | | 0.94 | < 0.05 |
| A3KGL9 | Non-histone chromosomal protein HMG-17 | | 0.95 | < 0.05 |
| Q9D281 | Protein Noxp20 (Nervous system overexpressed protein 20) | | 0.95 | < 0.05 |
| Q9CR98 | Protein FAM136A | | 0.95 | < 0.05 |
| Q69ZQ2 | Pre-mRNA-splicing factor ISY1 homolog | | 0.96 | 0.079 |
| Q80X50 | Ubiquitin-associated protein 2-like | | 0.96 | < 0.05 |
| A0A1L1SSH9 | Osteonectin (SPARC) | | 0.96 | < 0.05 |
| Q6S390 | Plectin 4 (Plectin 5) | | 0.97 | < 0.05 |
| Q61112 | 45 kDa calcium-binding protein (Cab45) | | 0.98 | < 0.05 |
| Q80U89 | MKIAA0034 protein (Fragment) | | 0.98 | 0.071 |
| Q80U88 | Eukaryotic translation initiation factor 4H (Fragment) | | 0.98 | < 0.05 |
| Q19LI2 | Alpha-1B-glycoprotein | | 0.98 | 0.067 |
| Q9CPT5 | Nucleolar protein 16 | | 0.98 | < 0.05 |
| P62869 | Elongin-B (EloB) | | 0.98 | < 0.05 |
| E9Q3E2 | Synaptopodin | | 0.98 | < 0.05 |
| Q9DAU1 | Protein canopy homolog 3 | | 0.99 | 0.052 |
| P38647 | Stress-70 protein | | 0.99 | < 0.05 |
| F6ZFU0 | Elongation factor 1-delta (Fragment) | | 0.99 | < 0.05 |
| Q9JMG1 | Endothelial differentiation-related factor 1 | | 1.00 | < 0.05 |
| Q60973 | Histone-binding protein RBBP7 | | 1.00 | < 0.05 |
| A0A0G2JFX7 | RNA-binding protein 8A | | 1.00 | < 0.05 |
| P63028 | Translationally-controlled tumor protein | | 1.00 | 0.085 |
| Q9D8Z2 | TP53-regulated inhibitor of apoptosis 1 | | 1.00 | < 0.05 |
| Q9JMG7 | Hepatoma-derived growth factor-related protein 3 (HRP-3) | | 1.00 | < 0.05 |
| Q9JJF0 | Nucleosome assembly protein 1-like 5 | | 1.01 | 0.081 |
| Q3TWZ9 | Clathrin light chain | | 1.01 | < 0.05 |
| Q9CZM2 | 60S ribosomal protein L15 | | 1.01 | < 0.05 |
| P97825 | Jupiter microtubule associated homolog 1 | | 1.02 | 0.064 |
| O08583 | THO complex subunit 4 | | 1.02 | < 0.05 |
| P12787 | Cytochrome c oxidase subunit 5A | | 1.03 | < 0.05 |
| Q9JHS9 | Spliceosome-associated protein CWC15 homolog | | 1.03 | < 0.05 |
| P54071 | Isocitrate dehydrogenase [NADP] | | 1.03 | < 0.05 |
| Q7TQH0 | Ataxin-2-like protein | | 1.03 | < 0.05 |
| Q9CR51 | V-type proton ATPase subunit G 1 | | 1.03 | < 0.05 |
| Q8R326 | Paraspeckle component 1 | | 1.04 | < 0.05 |
| Q8VDD5 | Myosin-9 | | 1.04 | 0.071 |
| E9PV24 | Fibrinogen alpha chain | | 1.04 | < 0.05 |
| Q3TIV5 | Zinc finger CCCH domain-containing protein 15 | | 1.04 | < 0.05 |
| Q62005 | Zona pellucida sperm-binding protein 1 | | 1.04 | < 0.05 |
| P99028 | Cytochrome b-c1 complex subunit 6 | | 1.04 | < 0.05 |
| A2AEW9 | GRIP1-associated protein 1 | | 1.05 | < 0.05 |
| P70699 | Lysosomal alpha-glucosidase | | 1.05 | < 0.05 |
| Q8BMK4 | Cytoskeleton-associated protein 4 | | 1.05 | < 0.05 |
| Q7TNE3 | Sperm-associated antigen 7 | | 1.05 | < 0.05 |
| Q6PFA2 | Clathrin light chain | | 1.05 | < 0.05 |
| Q60598 | Src substrate cortactin | | 1.05 | < 0.05 |
| Q3TS99 | PDZ domain-containing protein (Fragment) | | 1.06 | 0.053 |
| P47955 | 60S acidic ribosomal protein P1 | | 1.06 | < 0.05 |
| O08795 | Glucosidase 2 subunit beta | | 1.07 | < 0.05 |
| G5E866 | Splicing factor 3B subunit 1 | | 1.07 | < 0.05 |
| Q9JJU8 | SH3 domain-binding glutamic acid-rich-like protein | | 1.07 | < 0.05 |
| Q60932 | Voltage-dependent anion-selective channel protein 1 | | 1.08 | < 0.05 |
| E9Q7G0 | Nuclear mitotic apparatus protein 1 | | 1.08 | < 0.05 |
| Q99JI1 | Musculoskeletal embryonic nuclear protein 1 | | 1.08 | 0.092 |
| Q64314 | Hematopoietic progenitor cell antigen CD34 | | 1.08 | < 0.05 |
| P62077 | Mitochondrial import inner membrane translocase subunit Tim8 B | | 1.09 | < 0.05 |
| P52503 | NADH dehydrogenase [ubiquinone] iron-sulfur protein 6, mitochondrial | | 1.09 | < 0.05 |
| Q6LD55 | Apolipoprotein A-II | | 1.09 | < 0.05 |
| A0A2I3BRL8 | Predicted gene 7324 | | 1.09 | < 0.05 |
| E9QP00 | Transformer-2 protein homolog alpha | | 1.10 | < 0.05 |
| Q9CQR2 | 40S ribosomal protein S21 | | 1.10 | < 0.05 |
| B9VGS7 | Envelope protein | | 1.11 | < 0.05 |
| P57759 | Endoplasmic reticulum resident protein 29 | | 1.12 | < 0.05 |
| H3BLF8 | Isopentenyl-diphosphate Delta-isomerase | | 1.12 | 0.053 |
| Q9R0A0 | Peroxisomal membrane protein PEX14 | | 1.13 | < 0.05 |
| Q69ZC8 | GPALPP motifs-containing protein 1 | | 1.13 | < 0.05 |
| Q80UG5 | Septin-9 | | 1.14 | 0.073 |
| P62918 | 60S ribosomal protein L8 | | 1.15 | < 0.05 |
| O54998 | Peptidyl-prolyl cis-trans isomerase FKBP7 | | 1.15 | < 0.05 |
| Q3TWW8 | Serine/arginine-rich splicing factor 6 | | 1.15 | < 0.05 |
| Q8BI84 | Transport and Golgi organization protein 1 homolog | | 1.16 | 0.067 |
| Q02614 | SAP30-binding protein | | 1.16 | 0.093 |
| E9PUQ5 | Golgin subfamily A member 2 | | 1.16 | < 0.05 |
| Q923F1 | Chloride channel, nucleotide sensitive 1A | | 1.16 | < 0.05 |
| Q60950 | MYB-1a | | 1.16 | < 0.05 |
| A0A087WRY3 | Nuclear ubiquitous casein and cyclin-dependent kinase substrate 1 | | 1.16 | < 0.05 |
| Q91VL8 | Telomeric repeat-binding factor 2-interacting protein 1 | | 1.17 | < 0.05 |
| P47941 | Crk-like protein | | 1.17 | < 0.05 |
| G3X8T2 | Zinc finger CCCH domain-containing protein 18 | | 1.17 | < 0.05 |
| Q8CJ40 | Rootletin | | 1.18 | < 0.05 |
| O35685 | Nuclear migration protein nudC | | 1.18 | < 0.05 |
| Q5DTJ4 | MKIAA4178 protein (Fragment) | | 1.18 | < 0.05 |
| Q8BQ47 | Protein canopy homolog 4 | | 1.18 | < 0.05 |
| Q9Z315 | U4/U6.U5 tri-snRNP-associated protein 1 | | 1.18 | < 0.05 |
| Q8BFS6 | Serine/threonine-protein phosphatase CPPED1 | | 1.19 | < 0.05 |
| G5E898 | Periplakin | | 1.19 | 0.071 |
| O54724 | Caveolae-associated protein 1 | | 1.20 | < 0.05 |
| Q80W85 | Nucleoplasmin-2 | | 1.20 | < 0.05 |
| E9PXW9 | Transcription factor GATA-4 | | 1.20 | < 0.05 |
| P28654 | Decorin | | 1.20 | < 0.05 |
| P63158 | High mobility group protein B1 | | 1.21 | 0.080 |
| Q80TM2 | MKIAA1027 protein (Fragment) | | 1.21 | < 0.05 |
| Q3U6S1 | Vimentin | | 1.21 | < 0.05 |
| H7BX64 | Sarcolemmal membrane-associated protein | | 1.21 | < 0.05 |
| I6L958 | Igk protein | | 1.23 | 0.097 |
| Q80T68 | MKIAA3005 protein (Fragment) | | 1.24 | < 0.05 |
| P62843 | 40S ribosomal protein S15 | | 1.24 | < 0.05 |
| A0A0R4J0Q5 | Lamin-B2 | | 1.24 | < 0.05 |
| P60898 | DNA-directed RNA polymerase II subunit RPB9 | | 1.25 | < 0.05 |
| Q3UAI4 | SAP domain-containing protein | | 1.25 | < 0.05 |
| P70274 | Selenoprotein P (SeP) | | 1.25 | < 0.05 |
| Q9DCB8 | Iron-sulfur cluster assembly 2 homolog | | 1.25 | < 0.05 |
| P11404 | Fatty acid-binding protein | | 1.25 | 0.055 |
| E9Q9E1 | Eukaryotic translation initiation factor 4 gamma 1 | | 1.26 | < 0.05 |
| A0A498WFS2 | UBX domain-containing protein 1 | | 1.26 | < 0.05 |
| Q70IV5 | Synemin (Desmuslin) | | 1.26 | < 0.05 |
| F6XLV1 | Ciliary rootlet coiled-coil | | 1.26 | < 0.05 |
| Q8BGS2 | BolA-like protein 2 | | 1.27 | < 0.05 |
| P62830 | 60S ribosomal protein L23 | | 1.27 | < 0.05 |
| B1ARU4 | Microtubule-actin cross-linking factor 1 | | 1.27 | < 0.05 |
| Q99K28 | ADP-ribosylation factor GTPase-activating protein 2 | | 1.27 | < 0.05 |
| Q3TW77 | Uncharacterized protein | | 1.28 | < 0.05 |
| Q80WJ7 | Protein LYRIC (3D3/LYRIC) | | 1.28 | 0.066 |
| F6UK66 | Coiled-coil domain-containing protein 50 (Fragment) | | 1.29 | < 0.05 |
| A0A571BEI2 | AP2-associated protein kinase 1 (Fragment) | | 1.29 | < 0.05 |
| O88207 | Collagen alpha-1(V) chain | | 1.29 | < 0.05 |
| P19426 | Negative elongation factor E | | 1.30 | < 0.05 |
| Q6GTX3 | Apoe protein (Apolipoprotein E) | | 1.31 | < 0.05 |
| E9Q827 | cAMP-regulated phosphoprotein 19 | | 1.33 | < 0.05 |
| Q8K2T8 | RNA polymerase II-associated factor 1 homolog | | 1.34 | < 0.05 |
| Q9QZM0 | Ubiquilin-2 | | 1.34 | < 0.05 |
| Q99NB8 | Ubiquilin-4 | | 1.34 | < 0.05 |
| P11087 | Collagen alpha-1(I) chain | | 1.34 | < 0.05 |
| Q8C845 | EF-hand domain-containing protein D2 | | 1.35 | < 0.05 |
| P32020 | Non-specific lipid-transfer protein | | 1.36 | < 0.05 |
| P56873 | Protein ZNRD2 (Autoantigen p27 homolog) | | 1.36 | < 0.05 |
| Q3UDH7 | RRM domain-containing protein | | 1.37 | < 0.05 |
| P56375 | Acylphosphatase-2 | | 1.37 | < 0.05 |
| Q8VE97 | Serine/arginine-rich splicing factor 4 | | 1.37 | < 0.05 |
| Q9JHL1 | Na(+)/H(+) exchange regulatory cofactor NHE-RF2 (NHERF-2) | | 1.37 | < 0.05 |
| Q9D7S9 | Charged multivesicular body protein 5 | | 1.38 | < 0.05 |
| Q6ZWU9 | 40S ribosomal protein S27 | | 1.38 | < 0.05 |
| Q91VX2 | Ubiquitin-associated protein 2 | | 1.38 | < 0.05 |
| O89090 | Transcription factor Sp1 | | 1.38 | < 0.05 |
| H7BX95 | Serine/arginine-rich splicing factor 1 | | 1.39 | < 0.05 |
| Q6P5H2 | Nestin | | 1.39 | 0.056 |
| O70591 | Prefoldin subunit 2 | | 1.39 | < 0.05 |
| Q99LT0 | Protein dpy-30 homolog | | 1.40 | < 0.05 |
| Q5FWB6 | 60S acidic ribosomal protein P0 | | 1.40 | < 0.05 |
| Q9Z1M8 | Protein Red (Cytokine IK) | | 1.41 | 0.053 |
| P06728 | Apolipoprotein A-IV | | 1.42 | < 0.05 |
| Q00623 | Apolipoprotein A-I | | 1.42 | < 0.05 |
| Q9CWK8 | Sorting nexin-2 | | 1.42 | < 0.05 |
| Q9D0B6 | Protein PBDC1 | | 1.44 | < 0.05 |
| Q99K41 | EMILIN-1 | | 1.45 | < 0.05 |
| P43274 | Histone H1.4 | | 1.45 | 0.061 |
| Q9CQU0 | Thioredoxin domain-containing protein 12 | | 1.47 | < 0.05 |
| Q8CHG3 | GRIP and coiled-coil domain-containing protein 2 | | 1.49 | 0.084 |
| Q9D1J3 | SAP domain-containing ribonucleoprotein | | 1.49 | < 0.05 |
| P0C8B4 | EKC/KEOPS complex subunit GON7 | | 1.49 | < 0.05 |
| A0A286YDB7 | Signal sequence receptor subunit alpha | | 1.50 | < 0.05 |
| P08122 | Collagen alpha-2(IV) chain | | 1.52 | < 0.05 |
| A0A140LID0 | Probable global transcription activator SNF2L2 (Fragment) | | 1.52 | < 0.05 |
| E9PYH0 | Versican core protein | | 1.52 | < 0.05 |
| D3Z598 | Latent-transforming growth factor beta-binding protein 4 | | 1.52 | < 0.05 |
| Q6PDS4 | Frataxin intermediate form | | 1.53 | < 0.05 |
| Q3UKW1 | SERPIN domain-containing protein | | 1.54 | < 0.05 |
| G3UZM1 | Probable JmjC domain-containing histone demethylation protein 2C | | 1.55 | 0.069 |
| Q9DBL7 | Bifunctional coenzyme A synthase (CoA synthase) | | 1.55 | 0.069 |
| E9Q1P8 | Interferon regulatory factor 2-binding protein 2 | | 1.56 | < 0.05 |
| O88947 | Coagulation factor X | | 1.59 | < 0.05 |
| Q62241 | U1 small nuclear ribonucleoprotein C | | 1.59 | < 0.05 |
| Q8C5P7 | Testis development-related protein | | 1.60 | < 0.05 |
| Q62376 | U1 small nuclear ribonucleoprotein 70 kDa | | 1.60 | < 0.05 |
| Q14AS7 | Serine (Or cysteine) peptidase inhibitor | | 1.60 | 0.080 |
| Q9R1E0 | Forkhead box protein O1 | | 1.60 | < 0.05 |
| Q5M9P3 | 40S ribosomal protein S19 (Fragment) | | 1.61 | < 0.05 |
| Q9DCG9 | Multifunctional methyltransferase subunit TRM112-like protein | | 1.62 | < 0.05 |
| Q9CRB1 | Galectin | | 1.62 | < 0.05 |
| A0A1L7NR37 | Arg/Abl-binding protein 2 | | 1.63 | < 0.05 |
| A0A3B2WDD2 | Ribosomal protein | | 1.64 | < 0.05 |
| A3KMF2 | Mkl2 protein (Fragment) | | 1.65 | < 0.05 |
| A0A1W2P7A1 | 40S ribosomal protein S12 | | 1.66 | < 0.05 |
| Q9Z0F7 | Gamma-synuclein (Persyn) | | 1.66 | < 0.05 |
| A0A494BBA8 | 40S ribosomal protein S30 | | 1.67 | < 0.05 |
| Q6NZD2 | Sorting nexin-1 | | 1.67 | 0.062 |
| Q9WU28 | Prefoldin subunit 5 | | 1.67 | < 0.05 |
| P98078 | Disabled homolog 2 | | 1.67 | < 0.05 |
| Q8BHG9 | CGG triplet repeat-binding protein 1 | | 1.70 | < 0.05 |
| Q5XJE5 | RNA polymerase-associated protein LEO1 | | 1.73 | < 0.05 |
| A0A3B2WBC6 | DNA-directed RNA polymerase II subunit GRINL1A | | 1.74 | < 0.05 |
| E0CYH0 | Female-lethal(2)D homolog | | 1.75 | < 0.05 |
| Q01149 | Collagen alpha-2(I) chain | | 1.75 | < 0.05 |
| P10852 | 4F2 cell-surface antigen heavy chain | | 1.76 | < 0.05 |
| Q9CYA6 | Zinc finger CCHC domain-containing protein 8 | | 1.77 | < 0.05 |
| O08692 | Neutrophilic granule protein | | 1.79 | < 0.05 |
| Q3U4Y0 | H15 domain-containing protein | | 1.80 | < 0.05 |
| Q3TQI7 | Telomere length and silencing protein 1 homolog | | 1.80 | < 0.05 |
| Q3UIW3 | Uncharacterized protein | | 1.82 | < 0.05 |
| P62075 | Mitochondrial import inner membrane translocase subunit Tim13 | | 1.83 | < 0.05 |
| Q9WVA2 | Mitochondrial import inner membrane translocase subunit Tim8 A | | 1.83 | < 0.05 |
| Q9JHU2 | Palmdelphin | | 1.85 | 0.054 |
| Q9DC77 | Small muscular protein | | 1.87 | < 0.05 |
| P62073 | Mitochondrial import inner membrane translocase subunit Tim10 | | 1.89 | < 0.05 |
| P19001 | Keratin, type I cytoskeletal 19 | | 1.90 | < 0.05 |
| Q3UHU8 | General transcription factor II-I | | 1.91 | < 0.05 |
| T1ECW4 | RNA-binding protein with multiple-splicing | | 1.93 | < 0.05 |
| P31725 | Protein S100-A9 (Calgranulin-B) | | 1.93 | < 0.05 |
| P09528 | Ferritin heavy chain (Ferritin H subunit) | | 1.94 | 0.063 |
| Q61191 | Host cell factor 1 (HCF-1) | | 1.95 | < 0.05 |
| Q8R081 | Heterogeneous nuclear ribonucleoprotein L | | 2.01 | < 0.05 |
| Q3TRR0 | Microtubule-associated protein 9 | | 2.04 | < 0.05 |
| P43275 | Histone H1.1 (H1 VAR.3) | | 2.09 | < 0.05 |
| Q1WWK3 | Hist1h1b protein (Fragment) | | 2.10 | < 0.05 |
| Q3UYZ8 | Uncharacterized protein (Fragment) | | 2.10 | < 0.05 |
| Q8BHG2 | CXXC motif containing zinc binding protein | | 2.11 | < 0.05 |
| E9QAT4 | Protein transport protein Sec16A | | 2.12 | < 0.05 |
| Q9D0B0 | Serine/arginine-rich splicing factor 9 | | 2.12 | < 0.05 |
| Q8K003 | Translation machinery-associated protein 7 | | 2.13 | < 0.05 |
| Q3UMT1 | Protein phosphatase 1 regulatory subunit 12C | | 2.15 | < 0.05 |
| Q3UTI7 | Peptidyl-prolyl cis-trans isomerase | | 2.16 | < 0.05 |
| P47963 | 60S ribosomal protein L13 (A52) | | 2.17 | < 0.05 |
| P28653 | Biglycan (Bone/cartilage proteoglycan I) | | 2.19 | < 0.05 |
| Q3U962 | Collagen alpha-2(V) chain | | 2.23 | < 0.05 |
| Q8CH18 | Cell division cycle and apoptosis regulator protein 1 | | 2.24 | < 0.05 |
| A0A2I3BPG9 | Ribosomal protein L36A, pseudogene 1 | | 2.24 | < 0.05 |
| Q9CXZ1 | NADH dehydrogenase [ubiquinone] iron-sulfur protein 4 | | 2.25 | < 0.05 |
| Q03958 | Prefoldin subunit 6 (Protein Ke2) | | 2.26 | < 0.05 |
| Q3TWV0 | Vimentin | | 2.27 | < 0.05 |
| Q9EQC8 | Papillary Renal Cell carcinoma | | 2.27 | < 0.05 |
| Q9D823 | 60S ribosomal protein L37 | | 2.31 | < 0.05 |
| F2Z455 | Four and a half LIM domains protein 3 | | 2.32 | < 0.05 |
| Q9D0A3 | Arpin (Arp2/3 inhibition protein) | | 2.33 | < 0.05 |
| Q00899 | Transcriptional repressor protein YY1 | | 2.35 | < 0.05 |
| Q80U76 | Ribosome biogenesis regulatory protein (Fragment) | | 2.37 | < 0.05 |
| A0A140LJJ5 | A-kinase anchor protein 13 | | 2.43 | < 0.05 |
| Q99LP6 | GrpE protein homolog 1 | | 2.46 | < 0.05 |
| Q8CJF7 | Protein ELYS | | 2.53 | < 0.05 |
| Q9CQX8 | 28S ribosomal protein S36 | | 2.62 | < 0.05 |
| P47915 | 60S ribosomal protein L29 | | 2.64 | < 0.05 |
| P07309 | Transthyretin (Prealbumin) | | 2.67 | < 0.05 |
| P62900 | 60S ribosomal protein L31 | | 2.71 | < 0.05 |
| Q80YP5 | Integrin alpha 5 | | 2.71 | < 0.05 |
| P15864 | Histone H1.2 (H1 VAR.1) | | 2.72 | < 0.05 |
| O35215 | D-dopachrome decarboxylase | | 2.76 | 0.059 |
| E9QJT5 | Acylphosphatase | | 2.86 | < 0.05 |
| Q3UY34 | Protein CUSTOS | | 2.87 | < 0.05 |
| Q8R0P4 | Mth938 domain-containing protein (LI2) | | 2.96 | 0.089 |
| Q8VCG1 | Deoxyuridine 5'-triphosphate nucleotidohydrolase | | 2.97 | < 0.05 |
| Q3TLQ0 | Microtubule-associated protein | | 3.05 | < 0.05 |
| Q3U292 | H15 domain-containing protein | | 3.07 | < 0.05 |
| Q3UW40 | TRASH domain-containing protein | | 3.19 | < 0.05 |
| Q08093 | Calponin-2 | | 3.45 | < 0.05 |
| Q9ERU3 | Zinc finger protein 22 | | 3.79 | < 0.05 |
| Q99LN9 | Deoxyhypusine hydroxylase | | 6.10 | < 0.05 |
|  |  | |  |  |
