# Supplementary material for: Altered histone abundance as a mode of ovotoxicity during 7,12-dimethylbenz[a]anthracene exposure with additive influence of obesity
Source: Biol Reprod. 2023 Oct 19;110(2):419–29. doi: 10.1093/biolre/ioad140 (PMC10873273; doi:10.1093/biolre/ioad140)
Supplement: supplemental_table_7_100623_ioad140 [file supplemental_table_7_100623_ioad140.docx]

**Supplemental Table 7.** Female reproductive and DNA repair pathway proteins affected (*P* < 0.05; n = 5) by DMBA exposure in obese relative to lean mice.

| **Pathway** | **# of gene hits** | **Gene Names** | **Uniprot ID** |
| --- | --- | --- | --- |
| DNA replication (P00017) | 2 | Histone H3.3 | P84244 |
|  |  | Proliferating cell nuclear antigen | P17918 |
| Gonadotropin-releasing hormone receptor pathway (P06664) | 7 | Tubulin alpha-1B chain | P05213 |
|  |  | 45 kDa calcium-binding protein | Q61112 |
|  |  | Vinculin | Q64727 |
|  |  | Transcription factor Sp1 | O89090 |
|  |  | Caveolin-1 | P49817 |
|  |  | Y-box-binding protein 3 | Q9JKB3 |
|  |  | Integrin beta-1 | P09055 |
| p53 pathway (P00059) | 4 | Small ubiquitin-related modifier 1 | P63166 |
|  |  | 14-3-3 protein sigma | O70456 |
|  |  | High mobility group protein B1 | P63158 |
|  |  | Serine/threonine-protein phosphatase 2A catalytic subunit alpha isoform | P63330 |
| PI3 kinase pathway (P00048) | 2 | 14-3-3 protein zeta/delta | P63101 |
|  |  | Forkhead box protein O1 | Q9R1E0 |
