# Supplementary material for: Altered histone abundance as a mode of ovotoxicity during 7,12-dimethylbenz[a]anthracene exposure with additive influence of obesity
Source: Biol Reprod. 2023 Oct 19;110(2):419–29. doi: 10.1093/biolre/ioad140 (PMC10873273; doi:10.1093/biolre/ioad140)
Supplement: supplemental_figure_1_ioad140 [file supplemental_figure_1_ioad140.docx]

A


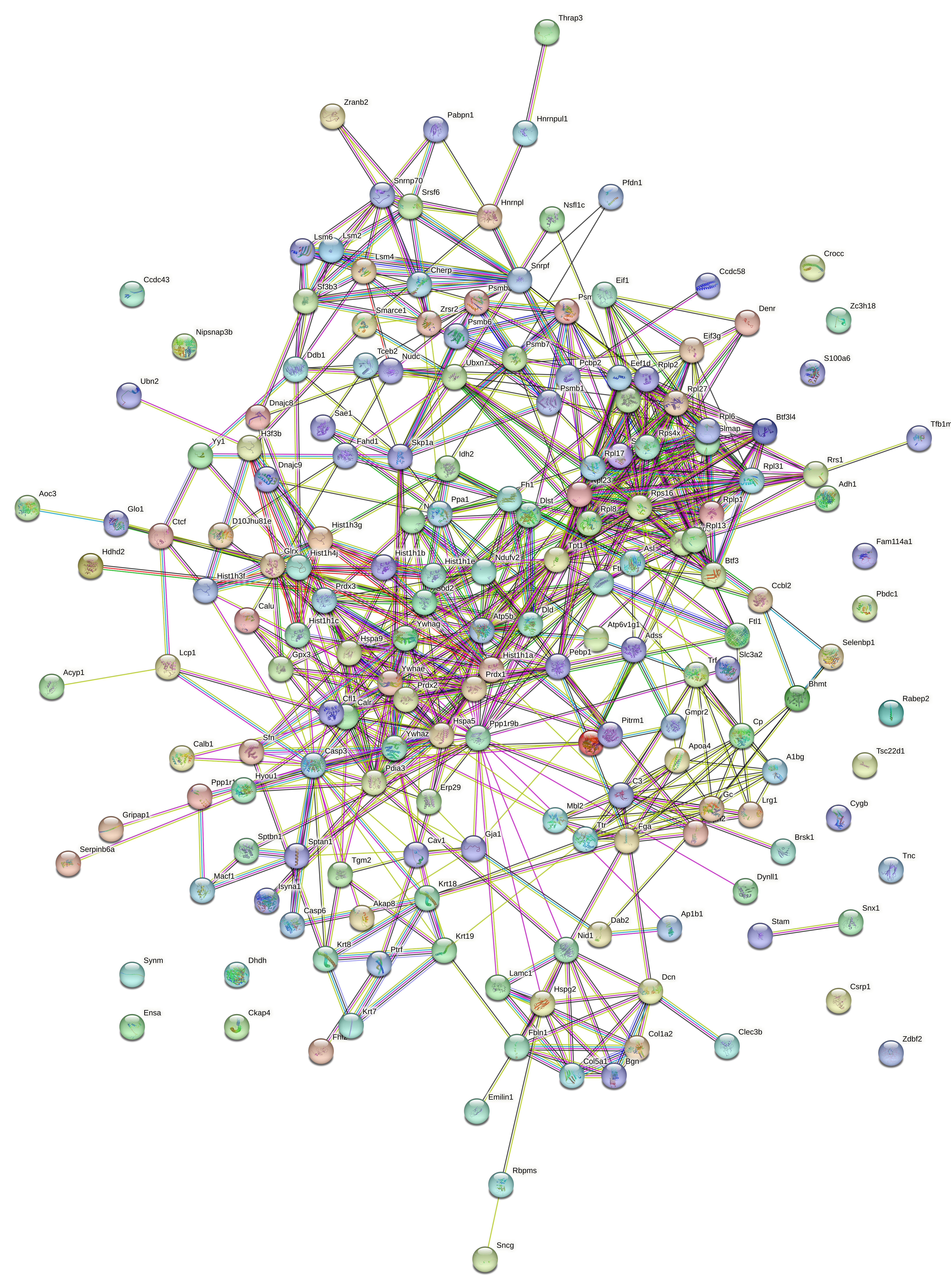


B


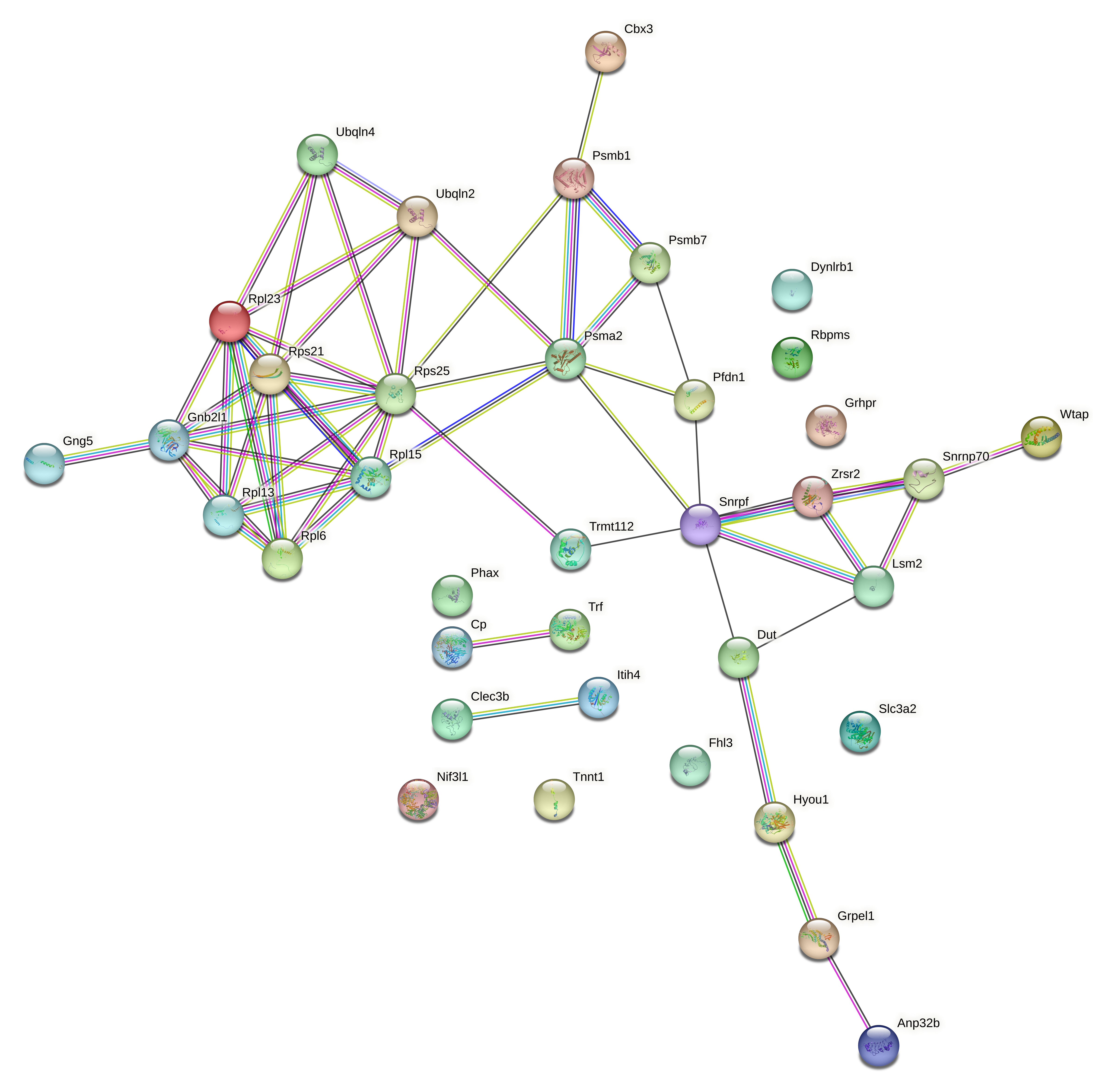


C


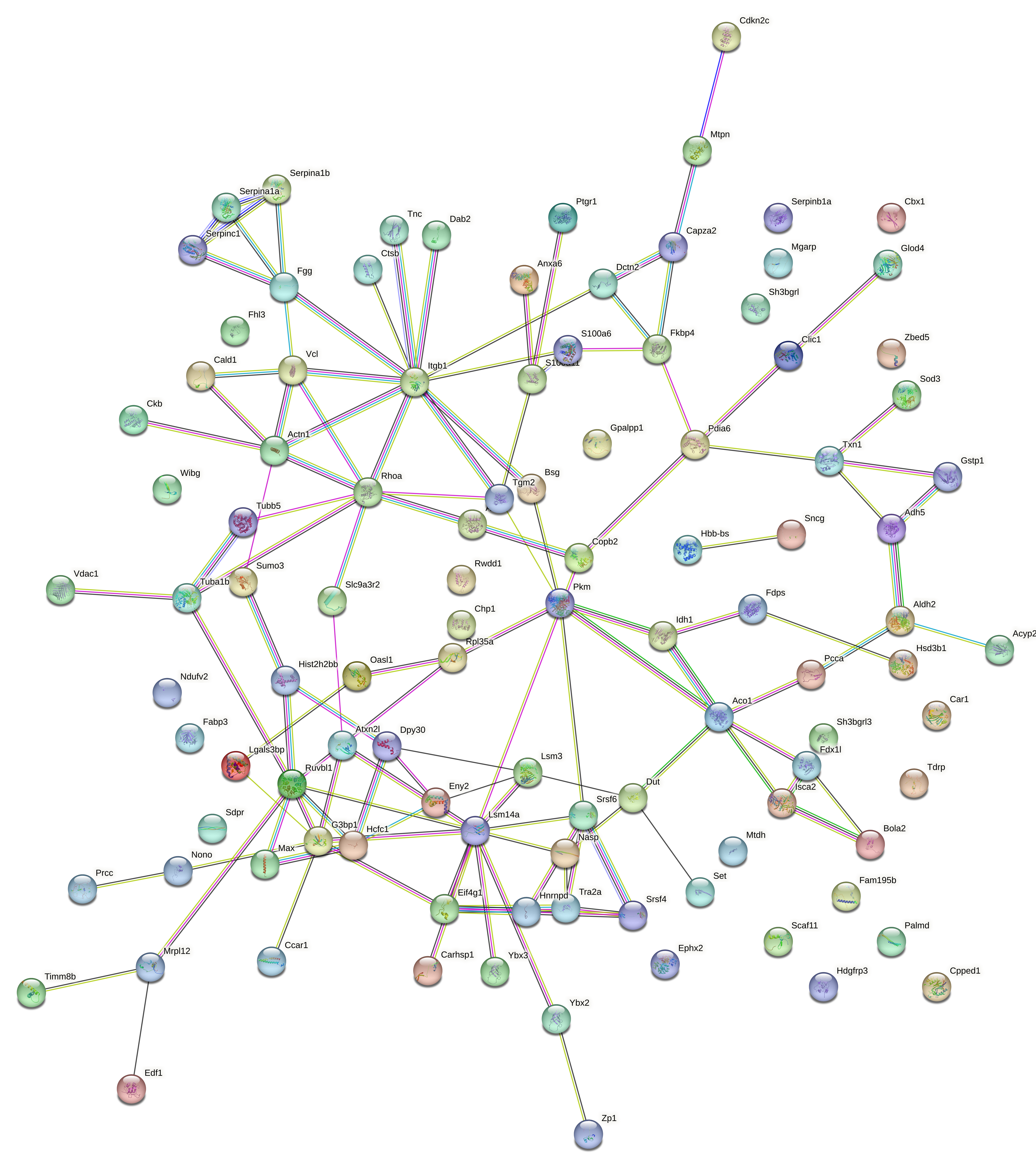


D

**Supplemental Figure 1.** Protein association networks of differentially expressed proteins in lean and obese mice ovaries due to DMBA. Functional protein-protein interaction networks are represented of proteins that were differentially expressed (*P* < 0.05; n = 5) due to A) obesity; B) DMBA exposure in lean mice; C) DMBA exposure in obese mice; and D) DMBA exposure in obese compared to lean mice.
